# Protein domain-dependent vesiculation of Lipoprotein A, a protein that is important in cell wall synthesis and fitness of the human respiratory pathogen *Haemophilus influenzae*

**DOI:** 10.3389/fcimb.2022.984955

**Published:** 2022-10-07

**Authors:** Farshid Jalalvand, Yu-Ching Su, Guillaume Manat, Alexey Chernobrovkin, Mahendar Kadari, Sandra Jonsson, Martina Janousková, Dorothea Rutishauser, Szabolcs Semsey, Anders Løbner-Olesen, Linda Sandblad, Klas Flärdh, Dominique Mengin-Lecreulx, Roman A. Zubarev, Kristian Riesbeck

**Affiliations:** ^1^ Clinical Microbiology, Department of Translational Medicine, Faculty of Medicine, Lund University, Malmö, Sweden; ^2^ Physiological Chemistry, Department of Medical Biochemistry and Biophysics, Karolinska Institute, Stockholm, Sweden; ^3^ Centre for Bacterial Stress Response and Persistence, Department of Biology, University of Copenhagen, Copenhagen, Denmark; ^4^ Department of Chemistry, Umeå University, Umeå, Sweden; ^5^ Department of Biology, Lund University, Lund, Sweden; ^6^ Institute for Integrative Biology of the Cell (I2BC), CEA, CNRS, Université Paris-Saclay, Gif-sur-Yvette, France

**Keywords:** Haemophilus influenzae, lipoprotein A, LpoA, outer membrane vesicles (OMV), respiratory pathogen

## Abstract

The human pathogen *Haemophilus influenzae* causes respiratory tract infections and is commonly associated with prolonged carriage in patients with chronic obstructive pulmonary disease. Production of outer membrane vesicles (OMVs) is a ubiquitous phenomenon observed in Gram-negative bacteria including *H. influenzae*. OMVs play an important role in various interactions with the human host; from neutralization of antibodies and complement activation to spread of antimicrobial resistance. Upon vesiculation certain proteins are found in OMVs and some proteins are retained at the cell membrane. The mechanism for this phenomenon is not fully elucidated. We employed mass spectrometry to study vesiculation and the fate of proteins in the outer membrane. Functional groups of proteins were differentially distributed on the cell surface and in OMVs. Despite its supposedly periplasmic and outer membrane location, we found that the peptidoglycan synthase-activator Lipoprotein A (LpoA) was accumulated in OMVs relative to membrane fractions. A mutant devoid of LpoA lost its fitness as revealed by growth and electron microscopy. Furthermore, high-pressure liquid chromatography disclosed a lower concentration (55%) of peptidoglycan in the LpoA-deficient *H. influenzae* compared to the parent wild type bacterium. Using an LpoA-mNeonGreen fusion protein and fluorescence microscopy, we observed that LpoA was enriched in “foci” in the cell envelope, and further located in the septum during cell division. To define the fate of LpoA, C-terminally truncated LpoA-variants were constructed, and we found that the LpoA C-terminal domain promoted optimal transportation to the OMVs as revealed by flow cytometry. Taken together, our study highlights the importance of LpoA for *H. influenzae* peptidoglycan biogenesis and provides novel insights into cell wall integrity and OMV production.

## Introduction

*Haemophilus influenzae* is a well-studied Gram-negative organism, and amoxicillin-resistant *H. influenzae* is one of the 12 most important pathogens on the global priority list of the World Health Organization (WHO, 2017). It is an evolutionarily close relative of *Escherichia coli* in the Gammaproteobacteria class, sharing a high degree of homology. However, it has a distinctly smaller genome (approx. 40% of *E. coli* K-12) that typically lacks redundant functional systems, also allowing an easier global study of the cellular content and functions. The bacterial species is divided into encapsular, *i.e.*, typeable *H. influenzae* and non-typeable *H. influenzae* (NTHi) that are capsule deficient. A vaccine exists against type b strains (Hib), and infections with those are nowdays a rare entity in most countries with proper child immunization programmes (Jalalvand and Riesbeck, 2018). Most cases of *H. influenzae* including invasive disease are thus nowdays associated with NTHi since a vaccine is not yet available. NTHi primarily causes upper respiratory tract infections in toddlers, acute otitis media, bronchitis, and exacerbations in patients with chronic obstructive pulmonary disease (COPD) (Jalalvand and Riesbeck, 2018; Su et al., 2018).

Gram-negative bacteria including *H. influenzae* produce extracellular outer membrane vesicles (OMVs; size 20-250 nm) that bud off from the outer membrane (OM), carrying lipopolysaccharides (LPS), membrane phospholipids, outer membrane proteins (OMPs), periplasmic content, and in some reported cases, genetic elements, and RNA (Unal et al., 2011; Schwechheimer and Kuehn, 2015; Sjostrom et al., 2015; McMillan and Kuehn, 2021; Orench-Rivera and Kuehn, 2021). Previous studies have implicated OMVs from *H. influenzae* in host-pathogen interactions, polymicrobial cooperation and antibiotic resistance (Sharpe et al., 2011; Schaar et al., 2014). *Haemophilus influenzae* OMVs also play a role in interactions with B cells (Deknuydt et al., 2014). Interestingly, vesicles derived from certain NTHi strains have the capability to activate human B cells *via* the IgD B cell receptor. OMVs from Gram-negative bacteria are also involved in biofilm formation and bacterial stress responses (Kaparakis-Liaskos and Ferrero, 2015; Schwechheimer and Kuehn, 2015), indicative of their various aspects in bacterial physiology. Furthermore, it has frequently been reported that certain envelope proteins are enriched or depleted in OMVs (Gill et al., 2019). Despite several hypothetic mechanisms have been suggested for this process, release of OMVs from Gram-negative bacteria is not yet fully elucidated (Bonnington and Kuehn, 2014).

The dynamic peptidoglycan (PG) homeostasis involves several effectors (synthases and hydrolases) and their cognate OM-located activators/inhibitors (Paradis-Bleau et al., 2010; Typas et al., 2010; Singh et al., 2015; Egan et al., 2017). Peptidoglycan is localized in the cell wall and surrounds most Gram-negative bacteria. It is also named the “sacculus” and dresses the cytoplasmic membrane preventing the cell from bursting due to turgor. The sacculus is composed of glycan chains, *i.e.*, a repeating unit of *N*-acetylglucosamine (GlcNAc) and *N*-acetylmuramic acid (MurNAc) fused with a β-1 to -4 linkage, and further interconnected by short peptides. PG is built from a disaccharide-peptide monomer unit that is attached to an undecaprenol lipid *via* a pyrophosphate linkage in lipid II. Once the lipid II precursor is transported to the outer face of the inner membrane, the disaccharide units are added to the growing glycan strands and cross-linked to the PG matrix by glycosyltransferases and transpeptidases, respectively. This PG assembly occurs *via* two different routes, either catalyzed by bifunctional class A penicillin-binding proteins, like PBP1A and PBP1B in *E. coli*, or by the combined action RodA or FtsW glycosyltransferases [belonging to the Shape, Elongation, Division, and Sporulation (SEDS) family] and cognate monofunctional transpeptidases like PBP2 or PBP3 (Daitch and Goley, 2020; Rohs and Bernhardt, 2021). Lipoprotein A (LpoA) and lipoprotein B (LpoB) are activators of PBP1A and PBP1B, respectively (Egan et al., 2020; Sardis et al., 2021). Recent years LpoA has been well described and crystallized both in *E. coli* and *H. influenzae* (Paradis-Bleau et al., 2010; Typas et al., 2010; Sathiyamoorthy et al., 2017; Kelley et al., 2019).

The goal of the present investigation was to study vesiculation and the role of LpoA in *H. influenzae*. Our data suggest that OMPs necessary for essential nutrient uptake are at the bacterial surface whereas proteins involved in host immune modulation and cell wall-synthesis regulation also are found in OMVs. Finally, we show that LpoA is concentrated in the OM prior to vesiculation, and that the C-terminal part contains a signal that appears to be important for transport into the OMVs.

## Material and methods

### Strains and culture conditions

A list of the strains and plasmids used in this study can be found in **Supplemental Table S1**. *H. influenzae* strains 3655 and Rd and isogenic mutants were routinely cultured on chocolate agar plates and in brain heart infusion (BHI) broth supplemented with NAD and hemin (10 µg/ml of each) (sBHI) as previously described (Unal et al., 2012). Kanamycin (30 µg/ml) was used for cultivation of *H. influenzae* strains carrying plasmids derived from pBZ485 (Harms et al., 2017). Conjugative A_2_pm-dependant *E. coli* JKE201 was cultured in LB liquid and solid medium supplemented with 1 mM A_2_pm (Sigma-Aldrich, Saint Louis, MO) as previously described (Harms et al., 2017). For time-lapse microscopy, bacteria were grown in ambient air at 37°C on solid transparent chemically defined medium (Herriott et al., 1970; Klein and Luginbuhl, 1979). Ciprofloxacin susceptibility was determined by Etest^®^ (Biomerieux, Durham, NC) on solid medium. Mecillinam (Sigma-Aldrich, Saint Louis, MO) susceptibility was determined by evaluation of overnight culture turbidity of bacteria inoculated in mecillinam-supplemented liquid media. OD_600nm_ measurements were conducted on a WPA BioWave CO8000 cell density meter (Biochrom, Cambridge, UK). Viable counts were performed by serial dilution of bacteria in PBS followed by spreading on chocolate agar plates. Colonies were counted after o/n incubation in 37°C using ProtoCOL 3 (SYNBIOSIS, Cambridge, UK).

### Isogenic mutants, plasmids, and recombinant protein

Plasmids used in this study are specified in **Supplemental Table S1**. Mutants were produced by transformation of competent *H. influenzae* as previously described (Jalalvand et al., 2013). Briefly, linear transformation vectors were produced using standard overlap polymerase chain reaction (PCR) in which *cat* (chloramphenicol acetyltransferase) was inserted between flanking regions to the targeted site. Mutations were verified with PCR and western blots. For deletion of *lpoA* (CGSHI3655_03836), *cat* was inserted immediately downstream of the endogenous *lpoA* promoter, replacing the open reading frame with the same start and stop codon, to not disrupt the additional two genes in the operon. We increased the incubation time for the isogenic mutant selection to 5 days instead of the conventional 48 h. The plasmids containing the genes for full length and truncated LpoA-mNeonGreen variants and P4-mCherry fusion were synthetically produced (GenScript Biotech, Piscataway, NJ) and cloned into pBZ485 between the *Sma*I and *Cla*I restriction sites (Harms et al., 2017). Plasmids were subsequently conjugated into *H. influenzae via* spotting of the *E. coli* donor strain (1:300 dilution of o/n liquid LB cultures) and the recipient strain (1:50 dilution of o/n liquid BHI culture) on BHI agar supplemented with A_2_pm (1 mM), hemin (10 µg/ml) and NAD (10 µg/ml). Conjugants were selected on chocolate agar plates containing 30 µg/ml kanamycin. For production of the isogenic Rd::*lpoA-mNeonGreen* and Rd::*omp_P4-mCherry* fusion strains, the constructs were cloned from the above-mentioned plasmids and fused with 1,000 base pairs (bp) from the 3’ end of the corresponding *lpoA* (locus tag HI1655) or *omp p4* gene (locus tag HI0693). The transformation vector was produced by overlap PCR inserting *cat* downstream of the reporter gene followed by 1,000 bp from the 5’ end of the flanking region. Recombinant full length mNeonGreen was produced *via* standard cloning into pET26b between the *Bam*HI and *Hin*dIII restriction sites. The open reading frame was amplified using pLpoA^1-576^-mNeonGreen as the template. The His-tagged protein was produced and affinity-purified as previously described (Jalalvand et al., 2013).

### Outer membrane isolation and OMV purification

Bacterial liquid cultures (500-1500 ml) were grown in Erlenmeyer flasks while shaking at 37°C. At indicated time points, cells were pelleted at 17,500*g* for 20 min at 4°C in a Sigma 6-16K centrifuge (Sigma-Aldrich). The OM was isolated from the pellet using *N*-lauroylsarcosine (sarkosyl) as described (Hobb et al., 2009). The supernatant was used for OMV purification. This fraction was passed through a Supor^®^-450 Gridded 0.45 nm filter (Pall Corporation, Ann Arbor, MI) to assure a cell-free sample. Subsequently, the supernatant was concentrated to 30 ml using VivaFlow 200 (100,000 MWCO) (Sartorius, Göttingen, Germany) and Masterflex L/S peristaltic pump (Cole-Palmer, Vernon Hills, IL). The concentrated sample was pelleted at 162,000x*g* for 1 h at 4°C in Sorvall Discovery M120 SE ultracentrifuge (Thermo Fisher Scientific, Waltham, MA). The OMV-containing pellet was resuspended in 60% Histodenz™ solution (Sigma-Aldrich) with 300 mM sucrose and 25 mM Tris-HCl. A Histodenz™ gradient ranging from 20% to 60% was layered on top of the sample using Hoefer™ SG Series Gradient Makers (Hoefer, Holliston, MA). The density-gradient ultracentrifugation was run at 200,000x*g* for 16-20 h at 4°C. Thereafter, the pure vesicle-containing fraction could be seen as a thin ring in the top half of the gradient tube. This was extracted, diluted 5x with ddH_2_O to remove the density-gradient buffer. Thereafter, OMVs were pelleted at 150,000x*g* for 1 h at 4°C. The pellet underwent one more round of washing with ddH_2_O before being resuspended in 200 µl PBS to yield purified vesicles. The protein concentration of the OMV samples were determined using Pierce™ BCA protein assay kit (Thermo Fisher Scientific, Waltham, MA). Sterility was checked by plating the final preparation on chocolate agar plates and growing plates overnight. Finally, to check the purity of OMV preparations and exclude that cell debris occurred we checked purified OMVs by Transmission electron microscopy (TEM) (**Supplemental Figure S1**) according to the protocol below.

### Nanosight nanoparticle tracking analysis

The sizes and concentrations of OMVs were determined using a Nanosight NS300 (Malvern Instruments, Malvern, UK) Nanoparticle Tracking Analysis (NTA) device, fitted with a 488 nm blue laser, a 20x lens and an EMCCD camera. All video interpretations were made in the accompanying software NTA 3.30 (Malvern). Before analysis, the OMV samples were diluted 1:1,000-1:100,000 in ultrapure PBS to avoid noise due to high concentration of nanoparticles. Each sample was analyzed at least three times and the results were averaged. Camera gain, thresholds and blur size were equally set for all samples.

### SDS-polyacrylamide gel electrophoresis (SDS-PAGE) and western blotting

Rabbit polyclonal α-mNeonGreen antibodies (pAb) was raised using recombinant mNeonGreen (Agrisera, Vännäs, Sweden). Thereafter, the total IgG from rabbit antiserum was purified using resin Hi-TrapTM Protein A FF (Cytiva, Marlborough, MA) according to manufacturer’s instruction. SDS-PAGE and western blotting was conducted according to standard protocol as previously described (Su et al., 2013). The α-mNeonGreen pAbs were used at 1:10,000 dilutions.

### Fluorescence microscopy

Phase contrast and fluorescence microscopy was performed using bacteria spotted on agarose pads, as previously described (Roghanian et al., 2019). For still images, bacteria were grown to late log phase in CDM containing 100 µM IPTG, diluted in PBS and transferred to agaros pads. Microscopy was performed using Olympus IX53 inverted fluorescence microscope, and the images were analyzed using the software Olympus cellSens Dimensions 1.18. For time-lapse microscopy, bacteria were scraped from o/n chocolate agar plates with 30 µM kanamycin, diluted in PBS and 2 µl were spotted on agarose pads containing CDM with 100 µM IPTG. The samples were transferred to the microscope enclosure and allowed to adjust to 37 °C for 30 min. A Zeiss Axio Observer.Z1 inverted light microscope with a motorized stage was used for this purpose as previously described (Frojd and Flardh, 2019). Zeiss ZEN 2.3 software was used for analysis of the images. Phase contrast and fluorescence images were recorded every 10 min for up to 6 h at 37 °C in temperature-controlled settings. All images were taken with the 100x objective in phase-contrast and fluorescent channels.

### Flow cytometry

Bacteria propagated in BHI broth and the corresponding OMV samples were harvested at indicated time points. Cells were washed three times in PBS to remove OMV contamination *via* centrifugation at 5,000 x *g*, 5 min, RT. The samples were then resuspended in PBS and, when needed, diluted 1:100-1:1,000. Flow cytometry analysis was performed on a BD FACSVerse Flow Cytometer (BD Biosciences, Franklin Lakes, NJ) equipped with a 488 nm laser and the data analyzed in the BD FACSuite software v1. Images were produced using FlowJo™ v10.8.1.

### Sample preparations for mass spectrometry

Sample pellets were dissolved in lysis buffer (0.1% ProteaseMax (PM)/5% AcN/150 mM AmBic). Samples were sonicated for 10 min in a water bath, followed by 1 min sonication with probe sonicator in pulse mode. Protein concentrations were measured using BCA assay. Equal amounts of protein from each sample (3 µg in 70 µL of 0.1% PM/5% AcN/50 mM AmBic) were sonicated once again for 10 min in a water bath and incubated for 30 min at 50°C. Proteins were reduced by adding 25 µl of 20 mM DTT in 50 mM AmBic for 30 min at 56°C, and then alkylated by adding 25 µl of 66 mM IAA in 50 mM AmBic for 30 min at room temperature. Proteins were digested by adding 25 µl of 13 ng/ul LC-grade trypsin (Promega, Madison, WI) and incubated overnight. After digestion, HFO was added (to 5%) and the plate was incubated for 30 min in 37°C. The samples were cleaned on a C-18 HyperSep plate and dried in a Savant Speedvac.

### Liquid chromatography-mass spectrometry (LC-MS/MS)

Chromatographic separation of peptides was achieved using in-house packed C18 column, 25 cm (Silica Tip 360 µm OD, 75 µm ID; New Objective, Woburn, MA) connected to the Ultimate™ 3000 nanoflow chromatography system (Thermo Scientific). Peptides were eluted at 300 nl/min flow rate for 120 min at a linear gradient from 2% to 26% ACN in 0.1% formic acid. The eluted peptides that were ionized with electrospray ionization were analyzed with Orbitrap QExactive Plus mass spectrometer (Thermo Fisher Scientific). The survey MS was acquired at the resolution of 120K in the range of m/z 200-2000. MS/MS data for 20 most intense precursors were obtained with a higher-energy collisional dissociation (HCD) for ions with charge z>1 at a resolution of 17,500.

### Proteomic data analysis

The mass spectrometric raw data were analyzed with the MaxQuant software (version 1.5.3.30). A false discovery rate (FDR) of 0.01 for proteins and peptides and a minimum peptide length of 6 amino acids were required. The Andromeda search engine was used to search the MS/MS spectra against the *H. influenzae* protein sequence database (Uniprot, 3087 sequences) combined with 262 common contaminants and concatenated with the reversed versions of all sequences. Enzyme specificity was set to trypsin, allowing cleavage N-terminal to proline. Peptide identification was based on a search with an initial mass deviation of the precursor ion of up to 7 ppm. The fragment m/z was set to 20 ppm. Only proteins quantified with at least two peptides were considered for quantitation. Analysis of the data provided by MaxQuant was performed in the R scripting and statistical environment. Normalization of the data was performed by equalizing the total ion signal in every analysis. Differences in relative protein abundances between treated and control samples were assessed by moderated *t*-test using limma package (Ritchie et al., 2015). Benjamini-Hochberg correction for multiple comparisons was used. PCA, *t*-test analysis and expression profile plots were performed using the log2-transformed protein abundance values. Outer membrane-associated proteins were identified in the literature or predicted by their subcellular localization using UniProtKB-HAMAP http://www.uniprot.org/).

### Scanning electron microscopy (SEM)

Bacteria were grown and concentrated in liquid broth, washed 3 times with distilled water for 10 min each, and fixed with 1% osmium in distilled water overnight. After washing off fixative with ddH^2^O (3 times for 30 min), bacteria were settled on coverslips covered with poly-L-lysine for 1 h. After settling, samples were dehydrated with increasing concentration of ethanol for 10 min each (70%, 80%, 90%, 95%, 100% twice) and dried with Leica EM CPD 300 Critical Point Dryer. Dried samples were glued on sample stubs with silver glue and coated with iridium (layer thickness: 5 nm) using Edwards S150A Dual Carbon/Sputter Coater. The samples were examined with Carl Zeiss Merlin Field Emission SEM operating with 4 kV and 150 pA at 20,000x and 50,000x magnification. Micrographs were recorded with SmartSEM V.5.05 software.

### High-pressure freezing and freeze substitution for transmission electron microscopy (TEM) imaging

Concentrated bacteria grown in liquid broth were directly filled into 3 nm carriers (Leica, Germany) and vitrified with Leica EM HMP100. Freeze substitution was carried out as previously described (Bleck et al., 2010). The infiltration at -90°C lasted for 48 h before the temperature was raised with 5°C per h until reaching -30°C. Samples were kept at -30°C for 3 h before the staining solution was washed out 3 times and replaced with acetone. The bacteria were infiltrated with increasing epoxy Embed812-DER 736 resin (EMS, Hatfield, PA) concentration (1:2. 1:1, 2:1 and 1:0) for 2 h each before final exchange to 100% epoxy resin overnight was done and polymerized at 60°C for 48 h. Epoxy resin embedded bacteria were sectioned with a Leica FC6 ultramicrotome and 45° diamond knife (Diatome, Nidau, Switzerland) into 80 nm thin sections, which were placed on formvar coated copper grids and counterstained with lead citrate. The samples were examined with a Jeol 1230 TEM operating at 80 kV at 40,000x magnification. Micrographs were recorded with Gatan Orius 830 with 2048 x 2048 pixels charge-coupled device (CCD) camera using the Digital Micrograph software.

### Isolation and quantitation of peptidoglycan

*Haemophilus influenzae* cultures (500 ml) were grown exponentially at 37°C in sBHI medium to an OD_600_ = 0.3. At this time, cultures were rapidly chilled to 4°C and cells were harvested. Bacteria were then rapidly suspended under vigorous stirring in 40 ml of a hot (95-100°C) aqueous 4% SDS solution for 1 h. After standing overnight at room temperature, the suspensions were centrifuged at 200,000 × *g* at 20°C for 20 min with a Beckman TL100 centrifuge and the resulting pellets, which contain the PG polymer, were washed several times with water. Final suspensions made in 2 ml of water were homogenized by brief sonication and aliquots were hydrolyzed (16 h at 95°C in 6 M HCl) and analyzed with a Hitachi model 8800 amino acid analyzer equipped with a 2620MSC-PS column (ScienceTec, Villebon-sur-Yvette, France). The fine structure of the PG polymer was then analyzed by the classical procedure described by Glauner and coworkers (Glauner, 1988; Glauner et al., 1988). The purified polymer (approx. 300 nmoles in terms of monomer) was digested overnight at 37°C in a reaction mixture (500 µl) containing 25 mM potassium phosphate buffer, pH 6.5, 0.25 mM MgCl_2_, lysozyme (200 µg) and mutanolysin (200 U). The released fragments (muropeptides) were reduced with sodium borohydride for 30 min and then separated by HPLC on a 3 µm ODS-Hypersil column (4.6 × 250 mm), using a gradient of methanol (from 0 to 25% in 100 min) in 50 mM sodium phosphate pH 4.5, at a flow rate of 0.5 ml in^-1^. Peaks were detected at 207 nm and the main muropeptides were collected for further characterization. A second HPLC step on the same column but using 0.05% trifluoroacetic acid and a gradient of methanol as eluent was performed for further purification and desalting of these compounds. The degree of crosslinkage in the PG polymer was defined as the percentage of crossbridges relative to the total number of disaccharide-peptide subunits (Glauner, 1988).

### General data analysis

If not specifically stated otherwise above, data points and graphs were processed and produced in Microsoft Excel for Mac v16.55 (Microsoft, Redmond, WA) and GraphPad Prism 9 (GraphPad Software, La Jolla, CA). The representation of LpoA crystal structure was produced in PyMOL 2.4.1 (http://pymol.org).

## Results

### The dynamics of *Haemophilus influenzae* 3655 vesiculation

**Figure 1:**
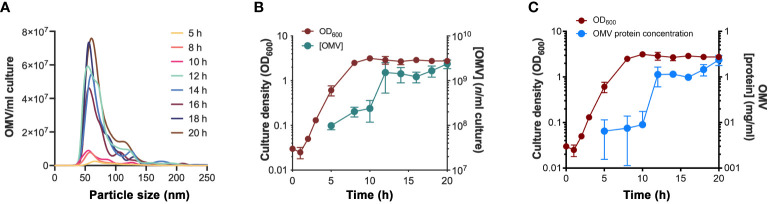
Vesiculation dynamics of *H. influenzae* at different growth phases. **(A)** Nanoparticle tracking analysis showing the size distribution of *H. influenzae* 3655 OMVs obtained at various growth phases. One replicate from three independent experiments is shown. **(B)** OD_600nm_ measurements of the growth of *H. influenzae* 3655 in liquid brain heart infusion (BHI) media and the corresponding levels of OMVs purified from the culture supernatant at indicated time points. **(C)** Protein concentration of the purified OMV samples in relation to the growth curve. The mean values and standard deviations from three independent experiments are plotted in panel **(B, C)**.

**Figure 2:**
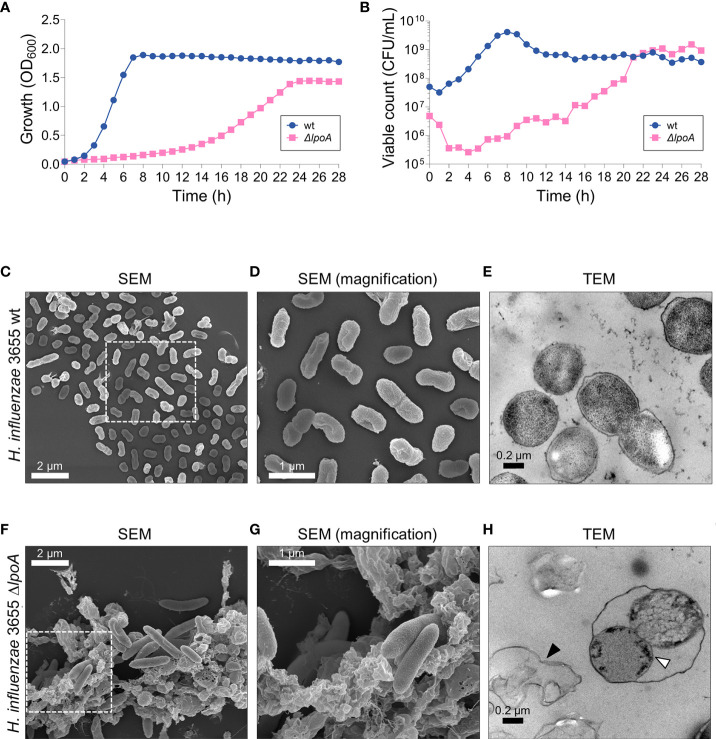
The loss of LpoA causes fitness defects in *H. influenzae.* **(A)** Measurements of the growth (culture OD_600_) and **(B)** viable count (CFU/ml) of *H. influenzae* 3655 wt and the Δ*lpoA* isogenic mutant revealed a dramatic loss of fitness associated with LpoA-deficiency. A representative experiment out of two is shown. **(C–H)** Electron microscopy images showing the morphological differences between wt **(C–E)** and the isogenic mutant Δ*lpoA* **(F–H)**. Scanning EM shows excessive cellular debris in the LpoA-deficient strain **(F)** compared to wt cells **(C)**. **(D, G)** Magnifications of the squared areas in **(C, F)** highlight the morphological differences between wt bacteria and the isogenic mutant. The phenotype is also observed in the TEM images with excessive cell debris prominently present around Δ*lpoA* cells (**H**; black arrow) but not the wt. Transmission EM also shows the OM detaching from dividing LpoA-deficient cells (white arrow). In **(C)** a representative area out of 6 is shown. In **(D)** a magnified area from **(C)** out of 8 areas with overview is shown. In **(F)** a representative area out of 5 is shown. In **(G)** a magnified area from **(F)** out of 9 areas with overview is shown. In **(E)** a representative area out of 16 examined is shown. In **(H)** a representative area out of 18 is shown. lpoA mutant **(F–H)**.

To discern the temporal dynamics of vesiculation in *H. influenzae*, we purified OMVs from different growth phases using density-gradient ultracentrifugation and determined the concentration and particle sizes using nanoparticle tracking analysis (NTA) (**Figures 1A, B**). Our results revealed that *H. influenzae* continuously produced OMVs, and that approximately 10-fold more vesicles occurred when bacteria entered the stationary phase. OMVs produced at different time points were comparably homogenous in size (means of 71.6-80.5 nm), except for samples obtained from the earliest time-point (5 h post-inoculation), which were significantly larger (mean 93.0 nm ± 66.1 nm SD) (**Figure 1A**, **Supplemental Table S2**). The protein concentration of purified OMV samples was determined, and we observed that concentrations correlated with the OMV quantity (**Figures 1B, C**). Between 10-12 h post-inoculation the OMV number increased dramatically, and the OMV concentration was highest upon entry into the stationary phase. Importantly, cell viability was unchanged during these time points (**Figure 2B**).

### Distribution of proteins in the OM and vesicles of *H. influenzae* 3655

To study the location of proteins in the two different compartments, we made a proteomic analysis of OMVs and the cellular OM using liquid chromatography–MS (LC-MS/MS). Vesicle preparations were obtained from overnight cultures (18 h) using multiple-step density-gradient ultracentrifugation and the cellular OM was extracted in the presence of Sarcosyl. Three independent replicate samples were prepared for each fraction and LC-MS/MS analyses were performed.

In total we identified 728 proteins that were present in both the OMV and OM samples. Lipoproteins as well as other well studied transmembrane proteins were represented in both the OM- and the OMV-associated OMPs. Our data is in agreement with previous reports (Sharpe et al., 2011; Michel et al., 2013); for instance, we observed that the PG-binding lipoprotein P6 (Pal), which anchors the OM to the PG network in the periplasm, was one of the most retained OMP on the bacterial surface and was underrepresented in the OMV fraction. Further, proteins involved in OM stability (Sharpe et al., 2011; Michel et al., 2013; Dong et al., 2014; Okuda et al., 2016) and nutrient uptake (Cope et al., 2000; Kemmer et al., 2001; Andersen et al., 2003; Morton et al., 2005) also mainly remained on the surface and were extracted from the OM with Sarcosyl. On the other hand, the OMVs contained LpoA and NlpI involved in cell wall-synthesis regulation and BamE and BamD from the β-barrel assembly machine (BAM)-complex (Hagan et al., 2011; Wu et al., 2014; Han et al., 2016; Lee et al., 2018). However, the essential component of the BAM pathway, BamA, was found in both the cell and OMVs. In addition, proteins involved in host immune modulation were in OMVs (Clementi et al., 2014; Tchoupa et al., 2015).

*Haemophilus influenzae* is auxotrophic for the essential co-factors heme and nicotinamide adenine dinucleotide (NAD), a rare trait that is clinically used to diagnose the pathogen (Jalalvand and Riesbeck, 2018). Our data show that all the major proteins involved in the uptake of NAD (P2 porin and OMP P4) and heme (HbpA, HgpB, HgpC and Hgp4) belonged to OMPs at the cellular surface (Cope et al., 2000; Kemmer et al., 2001; Andersen et al., 2003; Morton et al., 2005). Interestingly, we also observed that OMPs that were relatively more abundant in OMVs belonged to distinct functional categories that differed from the proteins retained in the OM. One category of proteins we found in OMVs was regulators of PG-synthesis/hydrolysis. NlpI, a protein that is involved in the modulation of PG endopeptidase activity (Singh et al., 2015), and previously implicated in OMV biogenesis (Schwechheimer et al., 2015), was also found in OMVs. The PG-synthase PBP1A-activator LpoA (Paradis-Bleau et al., 2010; Typas et al., 2010) was another lipoprotein that was in OMVs and was selected for downstream experiments.

### Lipoprotein A plays a critical role for *H. influenzae* fitness and PG homeostasis

Since LpoA was found in OMVs, we chose to investigate the fate of this lipoprotein. The rationale for selecting LpoA was two-fold: (i) the protein is highly interesting from a physiological standpoint, being critical for PG-synthesis, and (ii) its structure has been solved in *H. influenzae* (Vijayalakshmi et al., 2008; Sathiyamoorthy et al., 2017; Kelley et al., 2019). Despite LpoA is very important for PG-synthesis and has been reported to be essential in *H. influenzae* (Vijayalakshmi et al., 2008), we succeeded to delete *lpoA* in *H. influenzae* 3655 by ensuring that the remaining part of the operon was kept intact. The resulting isogenic *H. influenzae* Δ*lpoA* mutant had fitness defects as indicated by a reduced growth rate, but reached similar level of CFU at the stationary phase after 21 h as compared to the wild type bacterium (6 h) (**Figures 2A, B**). Moreover, the mutant exhibited a smaller colony size and was characterized by an increased susceptibility to the DNA gyrase/topoisomerase IV inhibitor ciprofloxacin that is transported through the membranes *via* porins (MIC <0.002 µg/ml compared to 0.008 µg/ml for the corresponding wild type [wt]) in addition to the PBP2 transpeptidase-inhibitor mecillinam (MIC 200 µg/ml and 400 µg/ml for Δ*lpoA* and wt, respectively) (data not shown).

To study the morphological phenotype of the deletion mutant, scanning- and transmission electron microscopy (SEM and TEM, respectively) were performed (**Figures 2C–H**). Micrographs revealed that isogenic Δ*lpoA* cells were elongated and surrounded by a considerable amount of cell debris (**Figures 2F, G**) compared to wt (**Figures 2C, D**). Using TEM, the OM of dividing LpoA-deficient cells was observed to be detached from the surface, suggesting a reduced stability of the cell envelope (**Figure 2H**). We observed that CFU count did not correlate with OD600 0.05 (**Figure 2B**), a fact that most likely was due to the difference in morphology.

Since LpoA has previously been shown to activate PG-crosslinking and glycan synthesis of PBP1a in *E. coli* (Paradis-Bleau et al., 2010; Typas et al., 2010; Sardis et al., 2021), we wanted to define whether the *H. influenzae* ortholog would exert the same function. The PG sacculus was extracted and purified from early log phase *H. influenzae* 3655 wt and isogenic Δ*lpoA*, and the amino acid and hexosamine contents of these extracts were determined after acid hydrolysis. Our results showed that the amount of PG in the *H. influenzae* Δ*lpoA* mutant cells was app. 55% lower as compared to that of the parental strain (**Table 1**).

**Table 1:** Peptidoglycan content of *H. influenzae* 3655 wt and isogenic Δ*lpoA* in early exponential phase.

Strain^a^	Conc. of indicated constituent in isolated sacculi (µmol/g of dry weight bacteria)			Mean value disaccharide peptide units^b^(µmol/g of dry weight bacteria)	Decrease compared to wt (%)
	Muramic acid	Glucosamine	meso-A_2_pm		
wt	7.4	6.9	7.0	7.1	-
Δ*lpoA*	3.3	3.2	3.1	3.2	54.7

^a^ *Haemophilus influenzae* and the corresponding ΔlpoA mutant were grown in the same conditions and harvested at different time points, but at the same optical density (OD 0.3) to avoid any interference with different growth phases. The final mass recovered per sample was 560.1 and 691.8 mg/L for NTHi 3655 and the corresponding ΔlpoA mutant, respectively.

^b^The amount of disaccharide peptide units of peptidoglycan corresponds to the average of the values for muramic acid, glucosamine and meso-A_2_pm.

**Figure 3:**
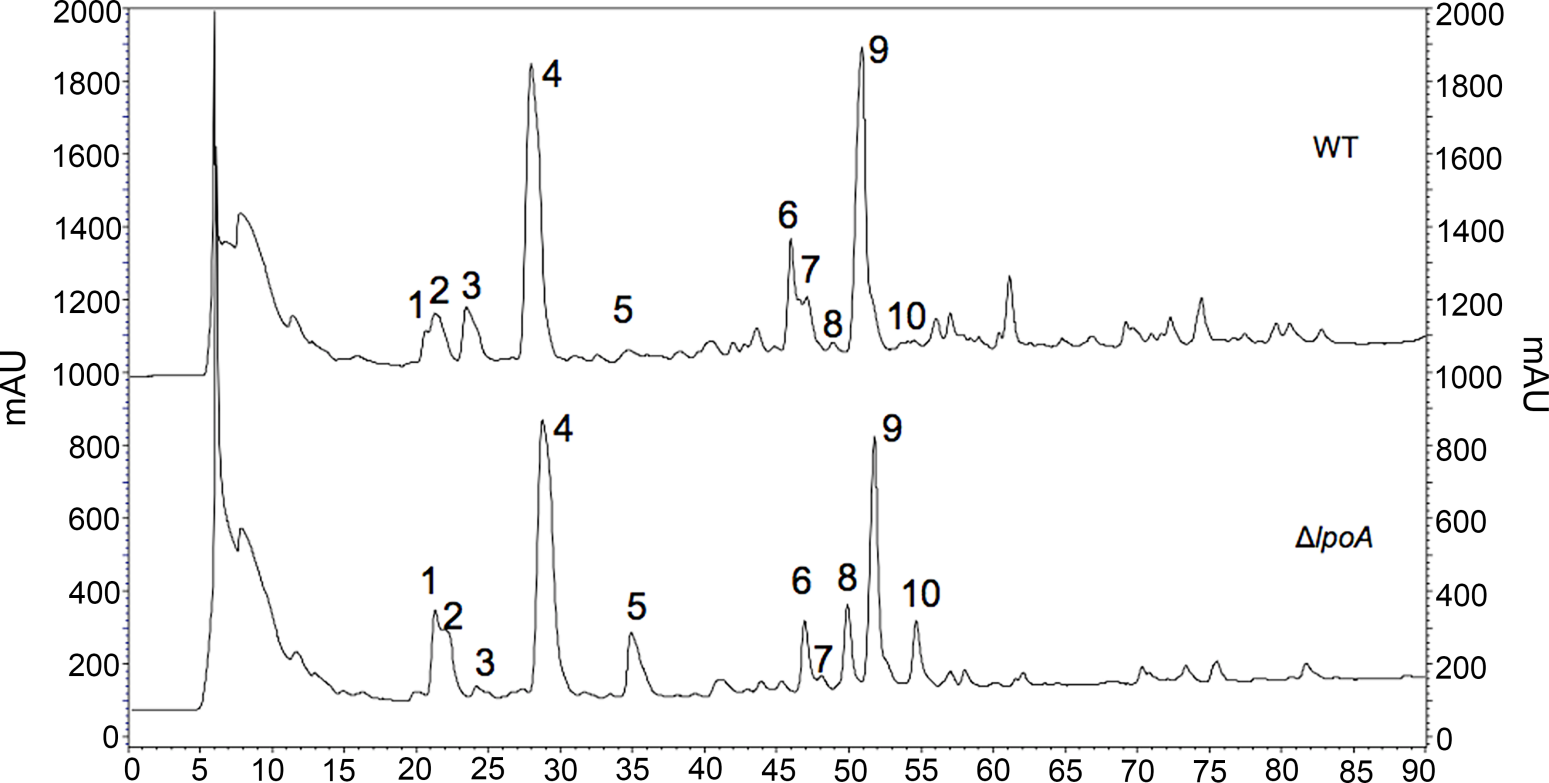
Aberrations in the cell wall of isogenic Δ*lpoA.* HPLC analysis of PG fragments (muropeptides) generated by digestion of PG from *H. influenzae* 3655 wt (upper panel) and Δ*lpoA* (lower panel) strains with muramidases (lysozyme and mutanolysin). Muropeptides were reduced by sodium borohydride and separated by HPLC. *mAU*, absorbance unit × 10^3^ at 207 nm. The identity of the main monomers (peaks 1-5) and dimers (peaks 6-10) was confirmed by amino acid and hexosamine composition analysis (see **Table 2**).

The fine structure of the PG was subsequently determined and found to significantly vary between wt and isogenic Δ*lpoA* (**Figure 3**, **Table 2**, and **Supplemental Figure S2**). The main aberrations observed in the Δ*lpoA* PG were: (i) an increased contribution of muropeptides carrying pentapeptide instead of tetrapeptide chains; (ii) a significant reduction of the proportion of dimers and thus of the degree of crosslinking in the polymer (**Table 2**). Indeed, when the main monomers and dimers that represent more than 90% of the PG structure were taken into consideration for this calculation, the degree of crosslinking was estimated to 23% and 17% in the *H. influenzae* wt and Δ*lpoA* mutant, respectively. The increased abundance of pentapeptide-containing muropeptides, which is consistent with the higher Ala/diaminopimelic acid (A_2_pm) ratio determined in the Δ*lpoA* mutant polymer, suggested a reduction of the activity of enzymes involved in PG crosslinking.

**Table 2:** Pattern of muropeptides identified by HPLC in *H. influenzae* 3655 wt and isogenic Δ*lpoA*.

Peak	Oligomer	Muropeptide	WT PG Amounts of muropeptides (nmoles)	% of total (b)	AlpoA PG Amounts of muropeptides (nmoles)	% of total (b)	Ratio AlpoA vs WT
1	monomer	GM-Tri	0.25	3.8	0.76	12.8	3.4
2 (a)	monomer	GM-Tetra(Asp)	0.18	2.7	0.05	0.85	0.32
2 (a)	monomer	GM-Tetra(Ser)	0.04	0.6	0.04	0.68	1.1
3	monomer	GM-Tetra(Gly)	0.31	4.7	0.07	1.18	0.25
4 (a)	monomer	GM-Tetra	2.71	41.2	2.25	37.8	0.92
4 (a)	monomer	GM-Penta(Gly)	0.04	0.6	0.41	6.9	11.5
5	monomer	GM-Penta -	0.06	0.91	0.37	6.22	6.8
6	dimer	GM-Tri/GM-Tetra	0.50	7.6	0.18	3.02	0.4
7	dimer	GM-Tetra/GM-Tetra(Gly)	0.26	3.95	0.06	1.0	0.25
8	dimer	GM-Tetra. / GM-Penta(Gly	< 0.02	< 0.3	0.27	4.55	> 15
9	dimer	GM-Tetra / GM-Tetra	2.19	33.3	1.34	22.5	0.68
10	dimer	GM-Tetra / GM-Penta	< 0.02	< 0.3	0.15	2.52	> 8

^a.^Muropeptides eluting at the same retention time in the classical conditions used for the separation of peptidoglycan muropeptides (**Figure S2**) were subsequently separated by a second HPLC step using TFA/methanol as elution system.

^b.^Respective contributions (in %) of individual muropeptides to the total amount of injected muropeptide mixture (about 6 nanomoles, in terms of A_2_pm content). Only the main monomers and dimers (peaks 1 to 10) were taken into consideration for this calculation.

### Lipoprotein A concentrates to foci in the *H. influenzae* OM

It has previously been reported that LpoA C-terminally fused to superfolder green fluorescent protein remains functional in *E. coli* (Paradis-Bleau et al., 2010). We therefore adopted the same strategy for *H. influenzae*, albeit exchanging the reporter to mNeonGreen (mNG) to ensure a monomeric conformation. In these experiments, we employed the *H. influenzae* model strain Rd (Fleischmann et al., 1995) for ectopic expression of LpoA-variants as the laboratory strain *H. influenzae* 3655 did not tolerate the different plasmids generated.

To study the subcellular location of LpoA, we constructed *H. influenzae* Rd LpoA^1-576^-mNG+P4^mcherry^. This strain co-expressed fusion proteins of LpoA-mNeonGreen and P4-mCherry. The expression of LpoA-mNG was continuously produced from the chromosome, whereby P4-mCherry was expressed from the plasmid pFJLU32 (pP4-mCherry) upon induction by isopropyl β-d-1-thiogalactopyranoside (IPTG) (**Supplemental Table S1**). P4 was selected for fusion with mCherry in this construct because it is one one of the OM lipoproteins that was not loaded in the OMV cargo. The visualization of P4-mCherry under the fluorescence microscope aided in “marking” the outer membrane layer on bacterial cells; whereby the cellular localization of LpoA was visualized *via* mNG. Of note, the strain did not exhibit any fitness defects associated with growth or viability (**Supplemental Figure S3**).

**Figure 4:**
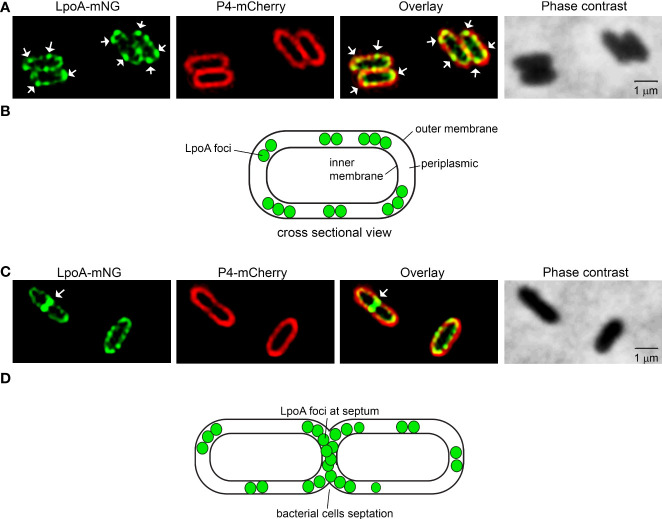
Fluorescence microscopy of *H. influenzae* expressing LpoA-mNeonGreen and P4-mCherry. **(A**, **C)** *H. influenzae* Rd pLpoA^1-576^ + P4^mcherry^ captured with phase contrast imaging and fluorescence channels detecting mNeonGreen and mCherry. Overlay of the fluorescence shown. White arrows indicate concentration of LpoA-mNeonGreen in foci in the OM **(A)** and at the septum **(C)**. White arrows indicate LpoA-mNeonGreen in foci in the OM. All images were taken at 100x magnification. Scale bars indicate 1 µM. **(B, D)** Cartoons indicating the localization of LpoA.

The LpoA-mNG-expressing *H. influenzae* Rd was subsequently grown to late log phase followed by P4-mCherry expression upon induction with IPTG. The purpose of this kinetics was to observe a strong mNG signal. The bacteria were then spotted on 1% agarose pads for fluorescence microscopy. Interestingly, we found that LpoA-mNG consistently concentrated into several foci, whereas P4-mCherry was evenly spread on the outer most layer of bacteria cells (**Figures 4A, B**). Moreover, LpoA-mNG was remarkly observed to be concentrated at the septum of dividing cells (**Figures 4C, D**), indicative of LpoA being involved in PG-synthesis during septal formation.

### C-terminal parts of LpoA are important for distribution in the OM and promotes translocation to OMVs

**Figure 5:**
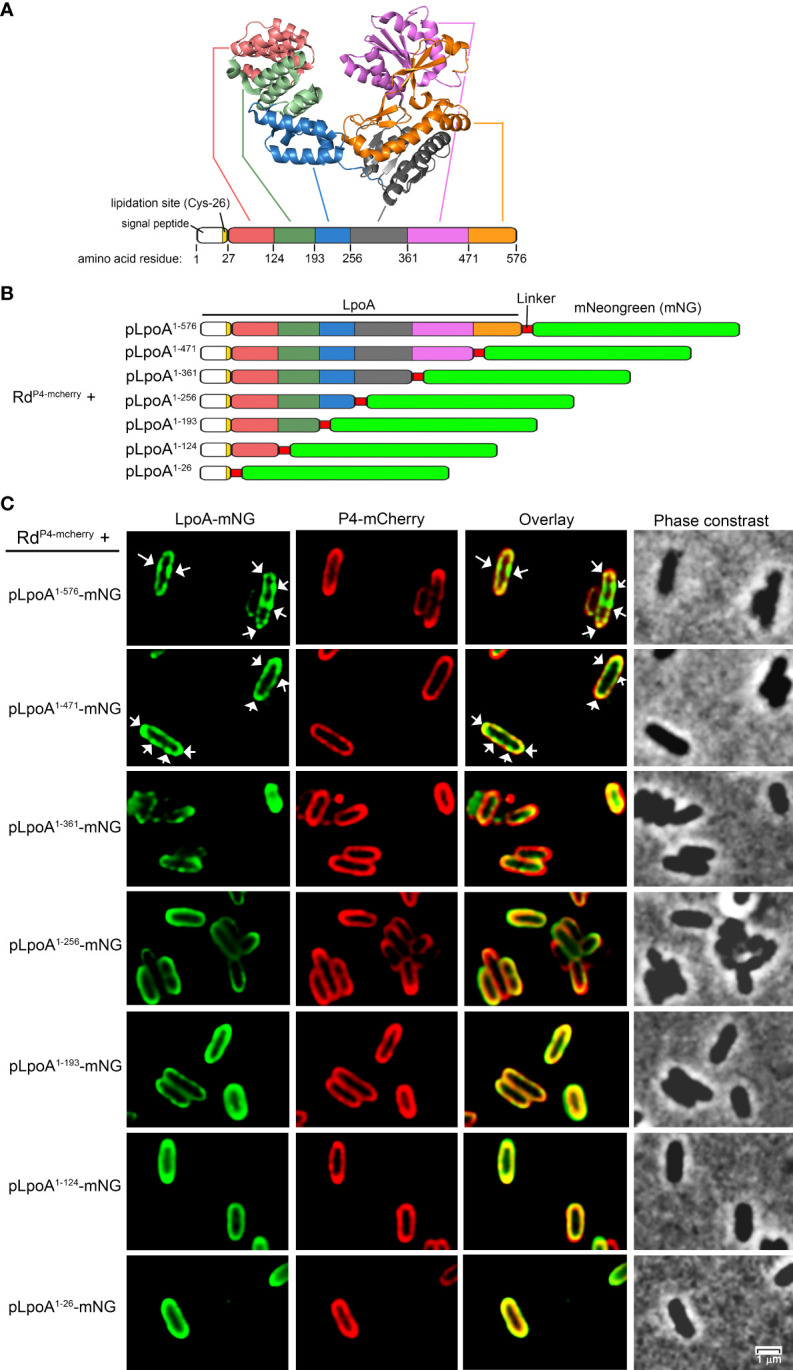
Fluorescence microscopy of *H. influenzae* simultaneously producing the outer membrane-retained OMP P4-mCherry different and various truncated LpoA-mNeonGreen variants. **(A)** Crystal structure of *H. influenzae* LpoA prepared in Pymol shows our arbitrary division of the lipoprotein into 6 domains and the corresponding amino acid residues. **(B)** Schematic representation of sequentially C-terminally truncated LpoA variants fused to mNeonGreen (mNG). The truncated LpoA constructs were introduced into the strain *H. influenzae* Rd P4^mcherry^ to produce the strains stated in the left column. **(C)** Phase contrast and fluorescence imaging of the strains indicated in panel **(B)**. White arrows indicate concentration of LpoA-mNG in foci in the OM **(C)**. All images were taken at 100x magnification. Scale bars indicate 1 µM.

To investigate whether a particular domain of LpoA was important for the protein distribution as foci in cell envelope or septum and hence influencing cellular behavior, we constructed, based on the published crystal structure (Vijayalakshmi et al., 2008; Sathiyamoorthy et al., 2017; Kelley et al., 2019), seven C-terminally truncated LpoA-variants fused to mNG (**Figures 5A, B**, **Table S1**). As LpoA is imperative for cellular fitness as shown by LpoA-defective mutant (**Figure 2**), we ectopically expressed this series of truncated forms of LpoA from plasmids in a strain that carried P4-mCherry chromosomally (*H. influenzae* Rd P4^mcherry^) (**Supplemental Table S1**). While full length LpoA^1-576^-mNG formed foci in the periplasmic compartment that was surrounded by OM (marked by P4-mCherry) (**Figure 5C**), our data indicated that the focus formation of LpoA gradually diminished with each truncated form until LpoA^1-256^-mNG was evenly spread in the periplasm. These data suggested that the C-terminal region of LpoA encompasing amino acid residues 257-576 might be involved in the formation of foci at the periplasm or at the septum of dividing cells. However, LpoA^1-471^-mNG and LpoA^1-361^-mNG were detected in the fluorescence microscope on the intact cell (**Figure 5C**), these two fusion proteins were found to be difficult to be detected by an antibody directed against mNG as revealed by western blots (**Supplemental Figure S4**).

**Figure 6:**
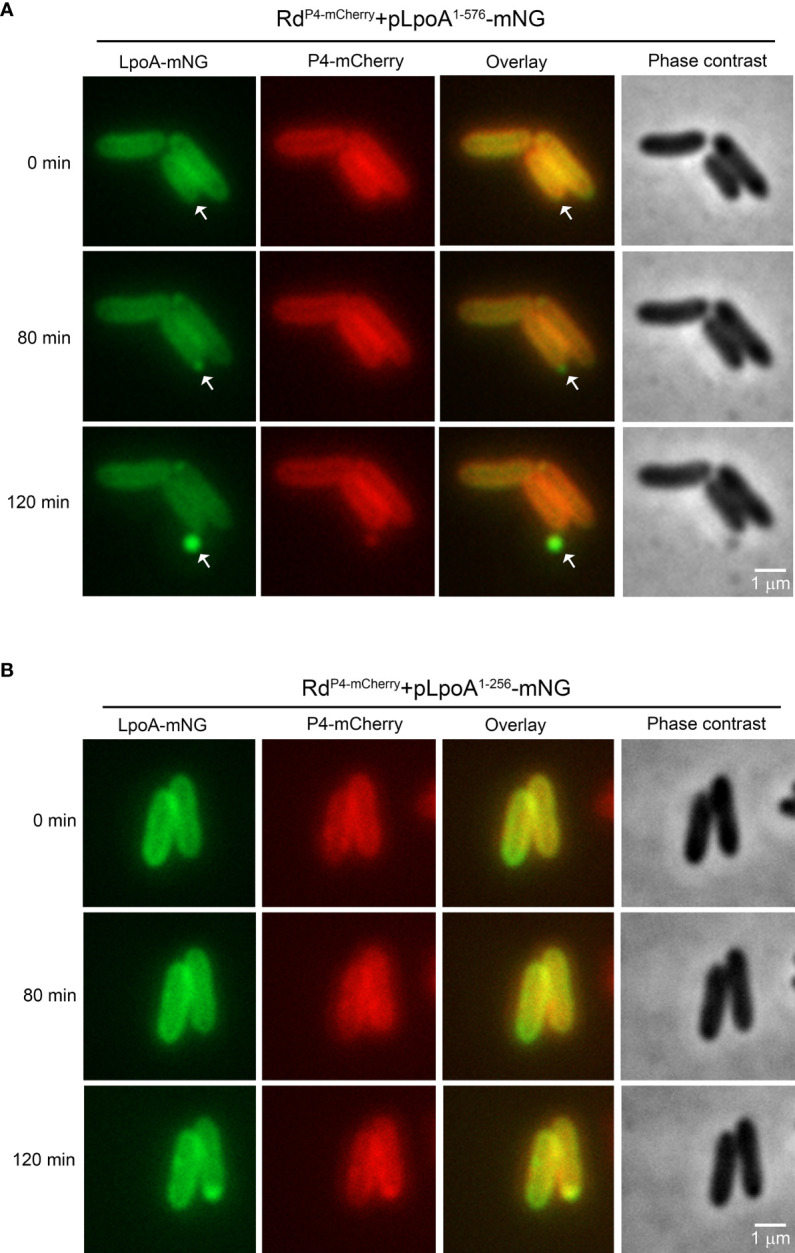
Time-lapse fluorescence microscopy of *H. influenzae* simultaneously producing the OM-retained P4-mCherry and two truncated LpoA-mNeonGreen variants. Bacteria were grown on solid CDM and induced by IPTG (100 µM). Phase contrast and fluorescence channel images were captured with 10 min intervals. **(A)** *H. influenzae* strain Rd P4^mcherry^+pLpoA^1-576^-mNG accumulating full length LpoA in foci prior to inclusion into OMVs (white arrows). **(B)** No fluorescent vesicles were released by *H. influenzae* Rd P4^mcherry^-pLpoA^1-256^-mNG expressing LpoA without the C-terminal (residues 257-576). All images were taken at 100x magnification. Scale bars indicate 1 µM.

To visualize how a C-terminal deletion in LpoA affected the translocation of this protein into vesicles, we performed time-lapse fluorescence microscopy under temperature-controlled settings with bacteria growing on solid nutrient for up to 6 h (**Figure 6**). Since the vesiculation dynamics changed depending on the growth phase, we resuspended bacterial colonies propagated on solid media for microscopy to capture bacteria in different growth phases. Interestingly, for Rd^P4-mCherry^+pLpoA^1-576^-mNG, we observed a continuous accumulation of LpoA-mNG in one cell pole over the time. OMVs that were visualized as green flouresence nanoparticle were finally produced at 120 minute and released at the cell pole (**Figure 6A**, **Supplemental Movie S1**). The green flourescense of OMVs in overlayed image indicated the presence of LpoA-mNG. However, outer membrane-located P4 that was fused with mCherry was not seen to the same extent as LpoA in the OMVs in the overlayed images, indicating a reduced level or absence of P4-mCherry in the vesicles. When C-terminal parts of LpoA (reside 257-576) were deleted (**Figure 6B**), we did not observe fluorescent OMVs as compared to that was seen with vesicles released by Rd^P4-mCherry^+pLpoA^1-576^-mNG (**Figure 6A**).

**Figure 7:**
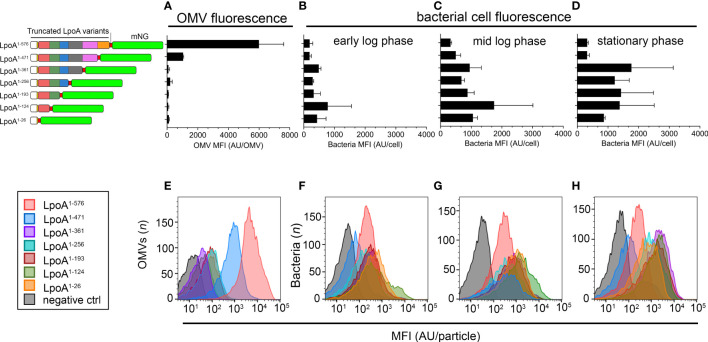
Quantification of single particle fluorescence in *H. influenzae* cells and OMVs. **(A–H)** We cultured *H. influenzae* Rd strains carrying plasmids with various truncated LpoA-mNeonGreen variants in liquid broth in the presence of IPTG (100 µM), starting with an inoculum of 5 x 10^7^ CFU/ml (OD_600nm_ = 0.03). Cell samples were then taken in early log phase (OD_600nm_ = 0.1), mid log phase (OD_600nm_ = 1) and stationary phase (o/n cultures, OD_600nm_ ≈ 3.0), washed thrice in PBS followed by measurement of cellular fluorescence. OMVs were isolated from the corresponding stationary phase cultures. **(A)** Measurement of mean fluorescence intensity (MFI) in OMVs purified from strains ectopically expressing the LpoA-mNG variants are shown to the left. **(B–D)** Accumulation of LpoA-mNG signals over the course of the growth curve in cells producing variants lacking the C-terminus. Culture conditions were as stated above. The mean and SD from two independent experiments are plotted. **(E–H)** One representative histogram from each experiment is shown.

To quantify the levels of LpoA-mNG in intact bacteria and OMVs in relation to C-terminal deletion in LpoA, we performed flow cytometry analysis on whole cells and OMVs (**Figures 7A–H**). We could only detect above background-level fluorescence in the OMVs produced by the Rd^P4-mCherry^ carrying pLpoA^1-576^-mNG (mean fluorescence intensity (MFI) 5,990 ± 1,621 arbitrary units (AU)) and pLpoA^1-471^-mNG (MFI 1,050 ± 30 AU) (**Figures 7A, E**). These findings indicated that C-terminal domains (residue 472-576) of LpoA might be important for inclusion of LpoA into OMVs, which was in good in agreement with the microscopy data (**Figure 6A**). A possible explanation for this could in fact be that the C-terminally truncated LpoA did not any longer associate with the bacterial membrane. In parallel, we observed that the mNG-signal accumulated in bacterial cells over the course of growth among mutants that produced LpoA variants with length up to residue 361. However, whole bacterial cell fluorescence remained low in strains expressing either full length LpoA (residue 1-576) or the C-terminal partially deleted LpoA (residue 1-471) (**Figures 7B–D, F–H**).

## Discussion

Outer membrane vesiculation is a hallmark of Gram-negative bacteria, and it has important physiological roles including host-pathogen interactions. Several studies have previously reported that OMV-dependent protein enrichment occurs in various bacterial species (Haurat et al., 2011; Elhenawy et al., 2014; Veith et al., 2014; Cahill et al., 2015; Schwechheimer & Kuehn, 2015; Veith et al., 2015). Our results revealed that *H. influenzae* (**Figures 1A–C**), like other species (Tashiro et al., 2010; Santos et al., 2012; Gerritzen et al., 2017), continuously produced OMVs, but that vesiculation increased approximately 10-fold when bacteria entered the stationary phase, most likely by accumulation of OMVs at the end of the culture. In contrast to almost all other bacterial species, *H. influenzae* lacks the biosynthetic enzymes needed to produce NAD and heme, and scavenges them from the mucosal environment of the host epithelium. Therefore, the bacteria seemed to exclude all important uptake systems from the OMV cargo.

An array of OM proteins was also found in *H. influenzae* OMVs as exemplified by P1, IgA protease, BamD and E, in addition to LpoA and NlpI. Since LpoA is extraordinary important for PG we focused our efforts on lipoprotein LpoA. *Haemophilus influenzae*, in analogy with *E. coli*, has two major bifunctional PG-synthases designated PBP1A and PBP1B. However, unlike *E. coli*, *H. influenzae* does not possess a homolog of LpoB, the essential activator of PBP1B in *Enterobacteriales* (Paradis-Bleau et al., 2010; Typas et al., 2010). This suggests that the two parallel OM lipoprotein-regulated PG-biosynthesis systems in *E. coli* are reduced to only one in *H. influenzae*, with LpoA being the key regulator. Although our LpoA mutant was viable it showed considerably defects and displayed decreased fitness. We show that, unlike the surface-retained OMP P4, which was evenly spread in the OM, LpoA in IPTG-induced bacteria was spatially concentrated to specific foci and display accumulation at septa from where vesiculation subsequently occurred (**Figures 4**, **5** and **Supplementary Movie 1**). This process was mediated by the LpoA C-terminus, potentially *via* protein-protein complex formation (Banzhaf et al., 2020). This contrasts to *E. coli*, that also carries LpoB; Typas *et al.* observed that LpoB is located in the septum, and LpoA for sidewall PG synthesis by interactions with PBP1B and PBP1A, respectively (Typas et al., 2010; Banzhaf et al., 2020). Since OMVs often are released from division septum during proliferation it has been shown that proteins localized at the septum can be enriched in OMVs; for example TolB in *Salmonella typhimurium* (Deatherage et al., 2009).

The PG profile of the wild type *H. influenzae* 3655 was also determined and carefully compared with the LpoA-deficient counterpart (**Figure 3**, **Table 2**, and **Supplemental Figure S2**). We interpret the results obtained as supportive for that *H. influenzae* LpoA exerts the same critical function for PG-synthesis as its *E. coli* ortholog (Paradis-Bleau et al., 2010; Typas et al., 2010; Sardis et al., 2021).

*Haemopilus influenzae* LpoA was found in OMVs as judged by protemics data. Moreover, fluorescence visualized endogenous LpoA expression using a *Haemophilus* strain carrying LpoA-mNeonGreen, and LpoA was located at the septum of dividing cells (**Figure 4**). It is noteworthy that LpoA has previously been observed to also be enriched in *Klebsiella pneumoniae* OMVs and is detected at high concentrations in *Vibrio cholerae* vesicles (Altindis et al., 2014; Cahill et al., 2015), which may suggest a general mechanism occurring in several species.

A limitation of our study is that we studied a series of truncated LpoA constructs using an IPTG-inducible expression system. It is a well-known fact that overexpressed proteins are enriched in OMVs. We also overexpressed full length non-tagged LpoA from a plasmid using the *lac*-promoter in *H. influenzae* Rd to study the effect on vesiculation (**Supplemental Figure S5**). Our data indicated that overexpression of LpoA caused a 12.7-fold increase of vesiculation. In line with this, Thoma *et al.* showed that overexpressed OM proteins are enriched in OMVs that can be harvested to study the particular OM proteins in more detail (Thoma et al., 2018). An interesting observation is that a further 10-fold increase in OMV production also occurs when *ompA* and *ompC* genes are deleted (Schwechheimer et al., 2014). Finally, another limitation of the present paper is that it was difficult to precisely determine the concentration of OMVs produced in the stationary phase since OMVs are accumulated during the whole culture period. In parallel, OMV production most likely had its maximum at 12 h, whereas harvest of OMVs were conducted at 18 h. To reduce the possible contamination with cell debris and OM, we used, however, a standardized protocol including Histodenz™ gradients (Lekmeechai et al., 2018) and checked for purity and homogeneity by TEM (**Supplemental Figure S1**). It needs to be noted that we solubilized the outer membrane from cells using Sarcosyl. This was intentionally done, intentionally done, but in the process we might have lost some proteins from the outer membrane since Sarcosyl may “pull out” also proteins from the outer membrane but not only dissociating proteins localized in the periplasm and the inner membrane (Chopra and Shales, 1980; Roier et al., 2016).

An obvious question was whether the amino acid residues (LpoA^362-576^) would itself be sufficient to transport mNG to OMVs. However, two separate constructs carrying the N-terminal lipoprotein signal sequence (LpoA^1-26^) with mNG fused either N- or C-terminally of the C-terminal LpoA-domain (LpoA^362-576^) failed to be exported out of the cytoplasm (**Supplemental Figure S6**). The accumulation of one of them in inclusion bodies suggested that the fusion proteins might have been sequestered.

To summarize, in parallel with other Gram-negative bacterial species, *H. influenzae* produces large amounts of OMVs, and a cargo-selection may occur. By studying LpoA in detail we determined that the C-terminal domain of LpoA is required for occurrence in OMVs. In line with mechanistic models for cargo-selection that previously have been suggested (Haurat et al., 2011; Elhenawy et al., 2014; Cahill et al., 2015; Ercoli et al., 2015; Schwechheimer and Kuehn, 2015; Veith et al., 2015; Roier et al., 2016), our study suggests that a protein domain-mediated mechanism may play a role in guiding LpoA to OMVs. However, additional factors such as chaperones might be required for mediating LpoA transport to OMVs. Further studies will thus be needed for better understanding of the complete process.

## Data availability statement

The datasets presented in this study can be found in online repositories. The names of the repository/repositories and accession number(s) can be found in the article/**Supplementary Material**.

## Author contributions

FJ planned and performed the majority of the experiments. AC, DR, and RZ conducted and analysed the proteomic experiments. GM and DM-L contrived, performed and analysed the peptidoglycan content experiments. MK contributed to strain construction. Y-CS contributed to the immunoblots analyses. NSB and LS generated the electron microscopy images. SS and AL-O contributed to the fluorescence microscopy experiments. KR and FJ conceived the project and analysed the data. FJ, Y-CS, and KR wrote the manuscript, DM-L contributed with the section on peptidoglycan analyses. All authors revised the manuscript. All authors read and approved the final manuscript.

## Funding

This work was supported by the Knut and Alice Wallenberg Foundation (to KR) Anna and Edwin Berger Foundation (KR), Swedish Heart Lung Foundation (KR; #20180401), the Royal Physiographical Society in Lund (to FJ; Forssman’s Foundation), the Skåne County Council’s research and development foundation (KR), and Swedish Research Council (KR; #2019-01053, KF; #2019-04643), the Novo Nordisk Foundation, the Danish National Research Foundation (FJ; DNRF120), and the Crafoord Foundation (FJ; 20190536, 2020542). The electron microscopy was conducted at the Umeå Centre for Electron Microscopy.

## Conflict of interest

The authors declare that the research was conducted in the absence of any commercial or financial relationships that could be construed as a potential conflict of interest.

## Publisher’s note

All claims expressed in this article are solely those of the authors and do not necessarily represent those of their affiliated organizations, or those of the publisher, the editors and the reviewers. Any product that may be evaluated in this article, or claim that may be made by its manufacturer, is not guaranteed or endorsed by the publisher.

## References

[B1] Altindis E. Fu Y. Mekalanos J. J. (2014). Proteomic analysis of vibrio cholerae outer membrane vesicles. Proc. Natl. Acad. Sci. United States America 111, E1548–E1556. doi: 10.1073/pnas.1403683111 PMC3992640 24706774

[B2] Andersen C. Maier E. Kemmer G. Blass J. Hilpert A. K. Benz R.. (2003). Porin OmpP2 of Haemophilus influenzae shows specificity for nicotinamide-derived nucleotide substrates. J. Biol. Chem. 278, 24269–24276. doi: 10.1074/jbc.M213087200 12695515

[B3] Banzhaf M. Yau H. C. Verheul J. Lodge A. Kritikos G. Mateus A.. (2020). Outer membrane lipoprotein NlpI scaffolds peptidoglycan hydrolases within multi-enzyme complexes in escherichia coli. EMBO J. 39, e102246. doi: 10.15252/embj.2019102246 32009249 PMC7049810

[B4] Bleck C. K. Merz A. Gutierrez M. G. Walther P. Dubochet J. Zuber B.. (2010). Comparison of different methods for thin section EM analysis of mycobacterium smegmatis. J. Microsc. 237, 23–38. doi: 10.1111/j.1365-2818.2009.03299.x 20055916

[B5] Bonnington K. E. Kuehn M. J. (2014). Protein selection and export *via* outer membrane vesicles. Biochim. Biophys. Acta 1843, 1612–1619. doi: 10.1016/j.bbamcr.2013.12.011 24370777 PMC4317292

[B6] Cahill B. K. Seeley K. W. Gutel D. Ellis T. N. (2015). Klebsiella pneumoniae O antigen loss alters the outer membrane protein composition and the selective packaging of proteins into secreted outer membrane vesicles. Microbiol. Res. 180, 1–10. doi: 10.1016/j.micres.2015.06.012 26505306

[B7] Chopra I. Shales S. W. (1980). Comparison of the polypeptide composition of *Escherichia coli* outer membranes prepared by two methods. J. Bacteriol. 144, 425–427. doi: 10.1128/jb.144.1.425-427.1980 6998960 PMC294674

[B8] Clementi C. F. Hakansson A. P. Murphy T. F. (2014). Internalization and trafficking of nontypeable Haemophilus influenzae in human respiratory epithelial cells and roles of IgA1 proteases for optimal invasion and persistence. Infection Immun. 82, 433–444. doi: 10.1128/IAI.00864-13 PMC3911862 24218477

[B9] Cope L. D. Hrkal Z. Hansen E. J. (2000). Detection of phase variation in expression of proteins involved in hemoglobin and hemoglobin-haptoglobin binding by nontypeable Haemophilus influenzae. Infection Immun. 68, 4092–4101. doi: 10.1128/IAI.68.7.4092-4101.2000 PMC101702 10858226

[B10] Daitch A. K. Goley E. D. (2020). Uncovering unappreciated activities and niche functions of bacterial cell wall enzymes. Curr. Biol. 30, R1170–r1175. doi: 10.1016/j.cub.2020.07.004 33022262 PMC7930900

[B11] Deatherage B. L. Lara J. C. Bergsbaken T. Rassoulian Barrett S. L. Lara S. Cookson B. T. (2009). Biogenesis of bacterial membrane vesicles. Mol. Microbiol. 72, 1395–1407. doi: 10.1111/j.1365-2958.2009.06731.x 19432795 PMC2745257

[B12] Deknuydt F. Nordström T. Riesbeck K. (2014). Diversion of the host humoral response: A novel virulence mechanism of Haemophilus influenzae mediated *via* outer membrane vesicles. J. Leukoc. Biol. 95, 983–991. doi: 10.1189/jlb.1013527 24550522

[B13] Dong H. Xiang Q. Gu Y. Wang Z. Paterson N. G. Stansfeld P. J.. (2014). Structural basis for outer membrane lipopolysaccharide insertion. Nature 511, 52–56. doi: 10.1038/nature13464 24990744

[B14] Egan A. J. Cleverley R. M. Peters K. Lewis R. J. Vollmer W. (2017). Regulation of bacterial cell wall growth. FEBS J. 284, 851–867. doi: 10.1111/febs.13959 27862967

[B15] Egan A. J. F. Errington J. Vollmer W. (2020). Regulation of peptidoglycan synthesis and remodelling. Nat. Rev. Microbiol. 18, 446–460. doi: 10.1038/s41579-020-0366-3 32424210

[B16] Elhenawy W. Debelyy M. O. Feldman M. F. (2014). Preferential packing of acidic glycosidases and proteases into bacteroides outer membrane vesicles. mBio 5, e00909–e00914. doi: 10.1128/mBio.00909-14 24618254 PMC3952158

[B17] Ercoli G. Tani C. Pezzicoli A. Vacca I. Martinelli M. Pecetta S.. (2015). LytM proteins play a crucial role in cell separation, outer membrane composition, and pathogenesis in nontypeable Haemophilus influenzae. mBio 6, e02575. doi: 10.1128/mBio.02575-14 25714719 PMC4358004

[B18] Fleischmann R. D. Adams M. D. White O. Clayton R. A. Kirkness E. F. Kerlavage A. R.. (1995). Whole-genome random sequencing and assembly of Haemophilus influenzae Rd. Science 269, 496–512. doi: 10.1126/science.7542800 7542800

[B19] Frojd M. J. Flardh K. (2019). Apical assemblies of intermediate filament-like protein FilP are highly dynamic and affect polar growth determinant DivIVA in streptomyces venezuelae. Mol. Microbiol. 112, 47–61. doi: 10.1111/mmi.14253 30929261

[B20] Gerritzen M. J. H. Martens D. E. Wijffels R. H. Stork M. (2017). High throughput nanoparticle tracking analysis for monitoring outer membrane vesicle production. J. Extracell Vesicles 6, 1333883. doi: 10.1080/20013078.2017.1333883 28717425 PMC5505008

[B21] Gill S. Catchpole R. Forterre P. (2019). Extracellular membrane vesicles in the three domains of life and beyond. FEMS Microbiol. Rev. 43, 273–303. doi: 10.1093/femsre/fuy042 30476045 PMC6524685

[B22] Glauner B. (1988). Separation and quantification of muropeptides with high-performance liquid chromatography. Anal. Biochem. 172, 451–464. doi: 10.1016/0003-2697(88)90468-X 3056100

[B23] Glauner B. Holtje J. V. Schwarz U. (1988). The composition of the murein of Escherichia coli. J. Biol. Chem. 263, 10088–10095. doi: 10.1016/S0021-9258(19)81481-3 3292521

[B24] Hagan C. L. Silhavy T. J. Kahne D. (2011). Beta-barrel membrane protein assembly by the bam complex. Annu. Rev. Biochem. 80, 189–210. doi: 10.1146/annurev-biochem-061408-144611 21370981

[B25] Han L. Zheng J. Wang Y. Yang X. Liu Y. Sun C.. (2016). Structure of the BAM complex and its implications for biogenesis of outer-membrane proteins. Nat. Struct. Mol. Biol. 23, 192–196. doi: 10.1038/nsmb.3181 26900875

[B26] Harms A. Liesch M. Korner J. Quebatte M. Engel P. Dehio C. (2017). A bacterial toxin-antitoxin module is the origin of inter-bacterial and inter-kingdom effectors of bartonella. PloS Genet. 13, e1007077. doi: 10.1371/journal.pgen.1007077 29073136 PMC5675462

[B27] Haurat M. F. Aduse-Opoku J. Rangarajan M. Dorobantu L. Gray M. R. Curtis M. A.. (2011). Selective sorting of cargo proteins into bacterial membrane vesicles. J. Biol. Chem. 286, 1269–1276. doi: 10.1074/jbc.M110.185744 21056982 PMC3020734

[B28] Herriott R. M. Meyer E. Y. Vogt M. Modan M. (1970). Defined medium for growth of Haemophilus influenzae. J. bacteriol. 101, 513–516. doi: 10.1128/jb.101.2.513-516.1970 5308770 PMC284935

[B29] Hobb R. I. Fields J. A. Burns C. M. Thompson S. A. (2009). Evaluation of procedures for outer membrane isolation from campylobacter jejuni. Microbiol. (Reading England) 155, 979–988. doi: 10.1099/mic.0.024539-0 PMC2763183 19246768

[B30] Jalalvand F. Riesbeck K. (2018). Update on non-typeable Haemophilus influenzae-mediated disease and vaccine development. Expert Rev. Vaccines 17, 503–512. doi: 10.1080/14760584.2018.1484286 29863956

[B31] Jalalvand F. Su Y. C. Morgelin M. Brant M. Hallgren O. Westergren-Thorsson G.. (2013). Haemophilus influenzae protein f mediates binding to laminin and human pulmonary epithelial cells. J. Infect. Dis. 207, 803–813. doi: 10.1093/infdis/jis754 23230060

[B32] Kaparakis-Liaskos M. Ferrero R. L. (2015). Immune modulation by bacterial outer membrane vesicles. Nat. Rev. Immunol. 15, 375–387. doi: 10.1038/nri3837 25976515

[B33] Kelley A. Vijayalakshmi J. Saper M. A. (2019). Crystal structures of the amino-terminal domain of LpoA from Escherichia coli and Haemophilus influenzae. Acta Crystallogr. F Struct. Biol. Commun. 75, 368–376. doi: 10.1107/S2053230X19004011 31045566 PMC6497104

[B34] Kemmer G. Reilly T. J. Schmidt-Brauns J. Zlotnik G. W. Green B. A. Fiske M. J.. (2001). NadN and e (P4) are essential for utilization of NAD and nicotinamide mononucleotide but not nicotinamide riboside in haemophilus influenzae. J. bacteriol. 183, 3974–3981. doi: 10.1128/JB.183.13.3974-3981.2001 11395461 PMC95280

[B35] Klein R. D. Luginbuhl G. H. (1979). Simplified media for the growth of Haemophilus influenzae from clinical and normal flora sources. J. Gen. Microbiol. 113, 409–411. doi: 10.1099/00221287-113-2-409 315998

[B36] Lee J. Sutterlin H. A. Wzorek J. S. Mandler M. D. Hagan C. L. Grabowicz M.. (2018). Substrate binding to BamD triggers a conformational change in BamA to control membrane insertion. Proc. Natl. Acad. Sci. United States America 115, 2359–2364. doi: 10.1073/pnas.1604100113 PMC5877925 29463713

[B37] Lekmeechai S. Su Y. C. Brant M. Alvarado-Kristensson M. Vallström A. Obi I.. (2018). Helicobacter pylori outer membrane vesicles protect the pathogen from reactive oxygen species of the respiratory burst. Front. Microbiol. 9, 1837. doi: 10.3389/fmicb.2018.01837 30245670 PMC6137165

[B38] McMillan H. M. Kuehn M. J. (2021). The extracellular vesicle generation paradox: A bacterial point of view. EMBO J. 40, e108174. doi: 10.15252/embj.2021108174 34636061 PMC8561641

[B39] Michel L. V. Snyder J. Schmidt R. Milillo J. Grimaldi K. Kalmeta B.. (2013). Dual orientation of the outer membrane lipoprotein P6 of nontypeable Haemophilus influenzae. J. bacteriol. 195, 3252–3259. doi: 10.1128/JB.00185-13 23687267 PMC3697637

[B40] Morton D. J. Madore L. L. Smith A. Vanwagoner T. M. Seale T. W. Whitby P. W.. (2005). The heme-binding lipoprotein (HbpA) of Haemophilus influenzae: role in heme utilization. FEMS Microbiol. Lett. 253, 193–199. doi: 10.1016/j.femsle.2005.09.016 16289530

[B41] Okuda S. Sherman D. J. Silhavy T. J. Ruiz N. Kahne D. (2016). Lipopolysaccharide transport and assembly at the outer membrane: The PEZ model. Nat. Rev. Microbiol. 14, 337–345. doi: 10.1038/nrmicro.2016.25 27026255 PMC4937791

[B42] Orench-Rivera N. Kuehn M. J. (2021). Differential packaging into outer membrane vesicles upon oxidative stress reveals a general mechanism for cargo selectivity. Front. Microbiol. 12, 561863. doi: 10.3389/fmicb.2021.561863 34276573 PMC8284480

[B43] Paradis-Bleau C. Markovski M. Uehara T. Lupoli T. J. Walker S. Kahne D. E.. (2010). Lipoprotein cofactors located in the outer membrane activate bacterial cell wall polymerases. Cell 143, 1110–1120. doi: 10.1016/j.cell.2010.11.037 21183074 PMC3085243

[B44] Ritchie M. E. Phipson B. Wu D. Hu Y. Law C. W. Shi W.. (2015). Limma powers differential expression analyses for RNA-sequencing and microarray studies. Nucleic Acids Res. 43, e47. doi: 10.1093/nar/gkv007 25605792 PMC4402510

[B45] Roghanian M. Semsey S. Lobner-Olesen A. Jalalvand F. (2019). (p)ppGpp-mediated stress response induced by defects in outer membrane biogenesis and ATP production promotes survival in Escherichia coli. Sci. Rep. 9, 2934. doi: 10.1038/s41598-019-39371-3 30814571 PMC6393671

[B46] Rohs P. D. A. Bernhardt T. G. (2021). Growth and division of the peptidoglycan matrix. Annu. Rev. Microbiol. 75, 315–336. doi: 10.1146/annurev-micro-020518-120056 34351794

[B47] Rohs P. D. A. Qiu J. M. Torres G. Smith M. D. Fivenson E. M. Bernhardt T. G. (2021). Identification of potential regulatory domains within the MreC and MreD components of the cell elongation machinery. J. Bacteriol. 203. doi: 10.1128/JB.00493-20 PMC8092158 33558391

[B48] Roier S. Zingl F. G. Cakar F. Durakovic S. Kohl P. Eichmann T. O.. (2016). A novel mechanism for the biogenesis of outer membrane vesicles in gram-negative bacteria. Nat. Commun. 7, 10515. doi: 10.1038/ncomms10515 26806181 PMC4737802

[B49] Santos S. Arauz L. J. Baruque-Ramos J. Lebrun I. Carneiro S. M. Barreto S. A.. (2012). Outer membrane vesicles (OMV) production of neisseria meningitidis serogroup b in batch process. Vaccine 30, 6064–6069. doi: 10.1016/j.vaccine.2012.07.052 22867717

[B50] Sardis M. F. Bohrhunter J. L. Greene N. G. Bernhardt T. G. (2021). The LpoA activator is required to stimulate the peptidoglycan polymerase activity of its cognate cell wall synthase PBP1a. Proc. Natl. Acad. Sci. U.S.A. 118. doi: 10.1073/pnas.2108894118 PMC8536351 34429361

[B51] Sathiyamoorthy K. Vijayalakshmi J. Tirupati B. Fan L. Saper M. A. (2017). Structural analyses of the Haemophilus influenzae peptidoglycan synthase activator LpoA suggest multiple conformations in solution. J. Biol. Chem. 292, 17626–17642. doi: 10.1074/jbc.M117.804997 28887305 PMC5663868

[B52] Schaar V. Uddback I. Nordstrom T. Riesbeck K. (2014). Group a streptococci are protected from amoxicillin-mediated killing by vesicles containing beta-lactamase derived from Haemophilus influenzae. J. antimicrobial. chemother. 69, 117–120. doi: 10.1093/jac/dkt307 23912886

[B53] Schwechheimer C. Kuehn M. J. (2015). Outer-membrane vesicles from gram-negative bacteria: biogenesis and functions. Nat. Rev. Microbiol. 13, 605–619. doi: 10.1038/nrmicro3525 26373371 PMC5308417

[B54] Schwechheimer C. Kulp A. Kuehn M. J. (2014). Modulation of bacterial outer membrane vesicle production by envelope structure and content. BMC Microbiol. 14, 324. doi: 10.1186/s12866-014-0324-1 25528573 PMC4302634

[B55] Schwechheimer C. Rodriguez D. L. Kuehn M. J. (2015). NlpI-mediated modulation of outer membrane vesicle production through peptidoglycan dynamics in Escherichia coli. Microbiologyopen 4, 375–389. doi: 10.1002/mbo3.244 25755088 PMC4475382

[B56] Sharpe S. W. Kuehn M. J. Mason K. M. (2011). Elicitation of epithelial cell-derived immune effectors by outer membrane vesicles of nontypeable Haemophilus influenzae. Infection Immun. 79, 4361–4369. doi: 10.1128/IAI.05332-11 PMC3257905 21875967

[B57] Singh S. K. Parveen S. SaiSree L. Reddy M. (2015). Regulated proteolysis of a cross-link-specific peptidoglycan hydrolase contributes to bacterial morphogenesis. Proc. Natl. Acad. Sci. United States America 112, 10956–10961. doi: 10.1073/pnas.1507760112 PMC4568209 26283368

[B58] Sjostrom A. E. Sandblad L. Uhlin B. E. Wai S. N. (2015). Membrane vesicle-mediated release of bacterial RNA. Sci. Rep. 5, 15329. doi: 10.1038/srep15329 26483327 PMC4612299

[B59] Su Y. C. Jalalvand F. Morgelin M. Blom A. M. Singh B. Riesbeck K. (2013). Haemophilus influenzae acquires vitronectin *via* the ubiquitous protein f to subvert host innate immunity. Mol. Microbiol. 87, 1245–1266. doi: 10.1111/mmi.12164 23387957

[B60] Su Y. C. Jalalvand F. Thegerström J. Riesbeck K. (2018). The interplay between immune response and bacterial infection in COPD: Focus upon non-typeable Haemophilus influenzae. Front. Immunol. 9,2530. doi: 10.3389/fimmu.2018.02530 30455693 PMC6230626

[B61] Tashiro Y. Ichikawa S. Shimizu M. Toyofuku M. Takaya N. Nakajima-Kambe T.. (2010). Variation of physiochemical properties and cell association activity of membrane vesicles with growth phase in pseudomonas aeruginosa. Appl. Environ. Microbiol. 76, 3732–3739. doi: 10.1128/AEM.02794-09 20382806 PMC2876431

[B62] Tchoupa A. K. Lichtenegger S. Reidl J. Hauck C. R. (2015). Outer membrane protein P1 is the CEACAM-binding adhesin of Haemophilus influenzae. Mol. Microbiol. 98, 440–455. doi: 10.1111/mmi.13134 26179342

[B63] Thoma J. Manioglu S. Kalbermatter D. Bosshart P. D. Fotiadis D. Müller D. J. (2018). Protein-enriched outer membrane vesicles as a native platform for outer membrane protein studies. Commun. Biol. 1, 23. doi: 10.1038/s42003-018-0027-5 30271910 PMC6123736

[B64] Typas A. Banzhaf M. van den Berg van Saparoea B. Verheul J. Biboy J. Nichols R. J.. (2010). Regulation of peptidoglycan synthesis by outer-membrane proteins. Cell 143, 1097–1109. doi: 10.1016/j.cell.2010.11.038 21183073 PMC3060616

[B65] Unal C. M. Schaar V. Riesbeck K. (2011). Bacterial outer membrane vesicles in disease and preventive medicine. Semin. Immunopathol. 33, 395–408. doi: 10.1007/s00281-010-0231-y 21153593

[B66] Unal C. M. Singh B. Fleury C. Singh K. Chavez de Paz L. Svensater G.. (2012). QseC controls biofilm formation of non-typeable Haemophilus influenzae in addition to an AI-2-dependent mechanism. Int. J. Med. Microbiol. 302, 261–269. doi: 10.1016/j.ijmm.2012.07.013 22954413

[B67] Veith P. D. Chen Y. Y. Chen D. O'Brien-Simpson N. M. Cecil J. D. Holden J. A.. (2015). Tannerella forsythia outer membrane vesicles are enriched with substrates of the type IX secretion system and TonB-dependent receptors. J. Proteome Res. 14, 5355–5366. doi: 10.1021/acs.jproteome.5b00878 26510619

[B68] Veith P. D. Chen Y. Y. Gorasia D. G. Chen D. Glew M. D. O'Brien-Simpson N. M.. (2014). Porphyromonas gingivalis outer membrane vesicles exclusively contain outer membrane and periplasmic proteins and carry a cargo enriched with virulence factors. J. Proteome Res. 13, 2420–2432. doi: 10.1021/pr401227e 24620993

[B69] Vijayalakshmi J. Akerley B. J. Saper M. A. (2008). Structure of YraM, a protein essential for growth of Haemophilus influenzae. Proteins 73, 204–217. doi: 10.1002/prot.22033 18412262

[B70] WHO (2017). WHO publishes list of bacteria for which new antibiotics are urgently needed. Available at: https://www.who.int/news/item/27-02-2017-who-publishes-list-of-bacteria-for-which-new-antibiotics-are-urgently-needed (Accessed September 21, 2022).

[B71] Wu S. Baum M. M. Kerwin J. Guerrero D. Webster S. Schaudinn C.. (2014). Biofilm-specific extracellular matrix proteins of nontypeable Haemophilus influenzae. Pathog. Dis 72(3):143–60. doi: 10.1111/2049-632X.12195 PMC4262604 24942343

