# Supplementary material for: Protein domain-dependent vesiculation of Lipoprotein A, a protein that is important in cell wall synthesis and fitness of the human respiratory pathogen Haemophilus influenzae
Source: Front Cell Infect Microbiol. 2022 Oct 7;12:984955. doi: 10.3389/fcimb.2022.984955 (PMC9585305; doi:10.3389/fcimb.2022.984955)
Supplement: Supplementary file 1 [file DataSheet_1.docx]

**Supplementary Fig. S1, Jalalvand *et al.***


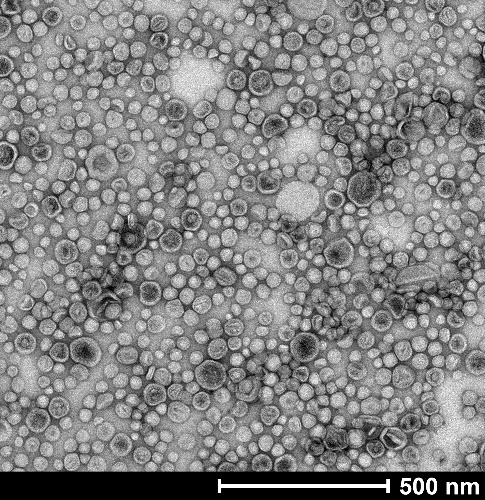


FIG S1 Transmission electron microscope visualization of isolated OMVs from NTHi 3655. Homogenous OMVs particles were seen, and debris cells could not be observed.
