# Supplementary material for: Protein domain-dependent vesiculation of Lipoprotein A, a protein that is important in cell wall synthesis and fitness of the human respiratory pathogen Haemophilus influenzae
Source: Front Cell Infect Microbiol. 2022 Oct 7;12:984955. doi: 10.3389/fcimb.2022.984955 (PMC9585305; doi:10.3389/fcimb.2022.984955)
Supplement: Supplementary file 2 [file DataSheet_2.docx]

**Supplementary Fig. S2, Jalalvand *et al.***


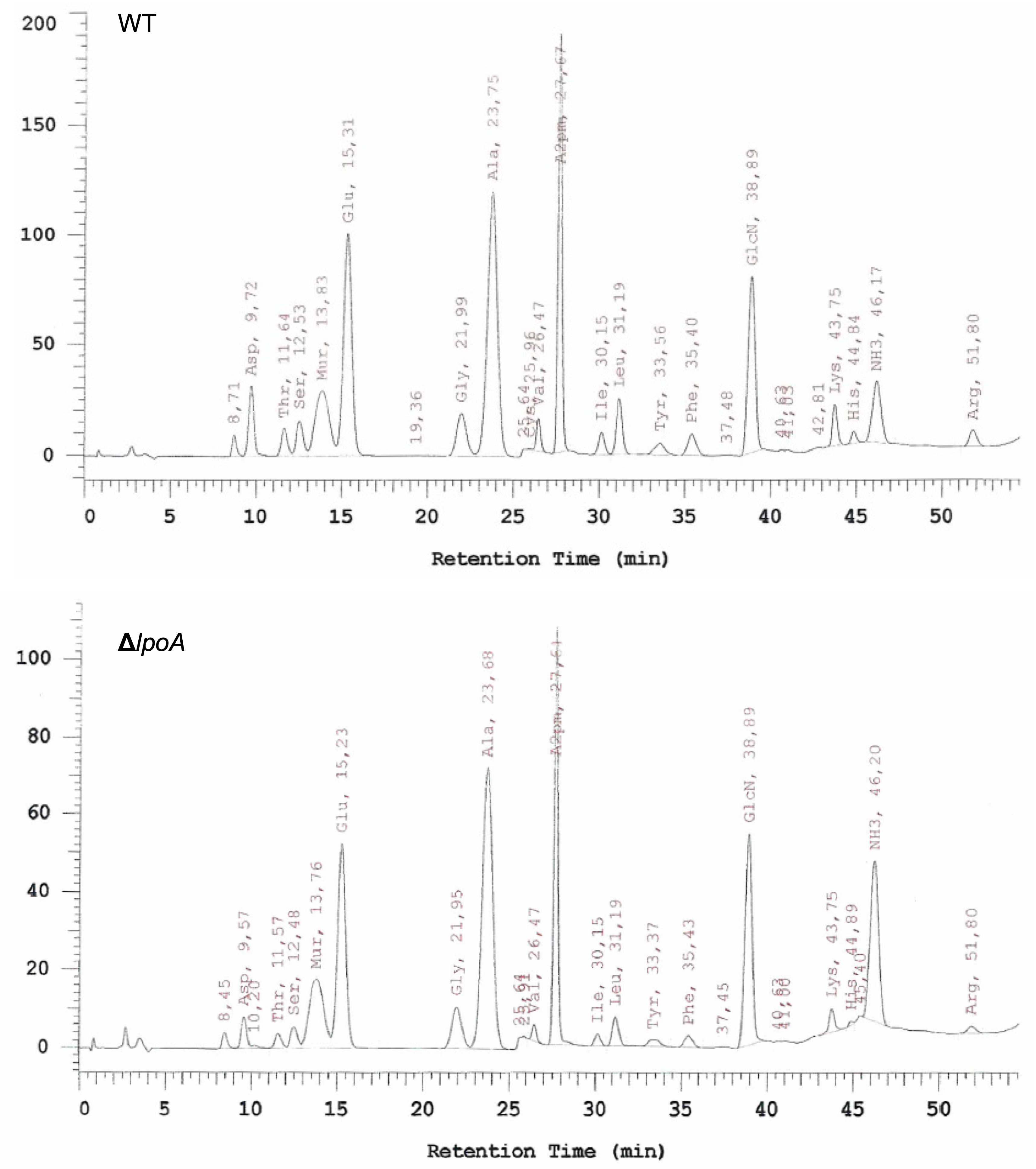


FIG. S2 Aminoacid and hexosamine composition of isolated peptidoglycan from *H. influenzae* wt and Δ*lpoA.* Aliquots of purified peptidoglycan from the two strains were hydrolyzed (16 h at 95°C in 6 M HCl) and the released amino acids and separated and quantitated with an amino acid analyzer. Mur, GlcN and A_2_pm correspond to muramic acid, glucosamine, and diaminopimelic acid, respectively, and other amino acids are represented by their 3-letter abbreviation.
