# Supplementary material for: Protein domain-dependent vesiculation of Lipoprotein A, a protein that is important in cell wall synthesis and fitness of the human respiratory pathogen Haemophilus influenzae
Source: Front Cell Infect Microbiol. 2022 Oct 7;12:984955. doi: 10.3389/fcimb.2022.984955 (PMC9585305; doi:10.3389/fcimb.2022.984955)
Supplement: Supplementary file 3 [file DataSheet_3.docx]

**Supplementary Fig. S3, Jalalvand *et al.***





FIG S3 No difference in growth fitness of *H. influenzae* Rd LpoA^1-576^-mNG+P4^mcherry^ compared to *H. influenzae* Rd with and without induction by IPTG. Two typical experiments are shown in panels a to d. The experiments were done with a Tecan Spark.
