# Supplementary material for: Protein domain-dependent vesiculation of Lipoprotein A, a protein that is important in cell wall synthesis and fitness of the human respiratory pathogen Haemophilus influenzae
Source: Front Cell Infect Microbiol. 2022 Oct 7;12:984955. doi: 10.3389/fcimb.2022.984955 (PMC9585305; doi:10.3389/fcimb.2022.984955)
Supplement: Supplementary file 4 [file DataSheet_4.docx]

**Supplementary Fig. S4, Jalalvand *et al.***


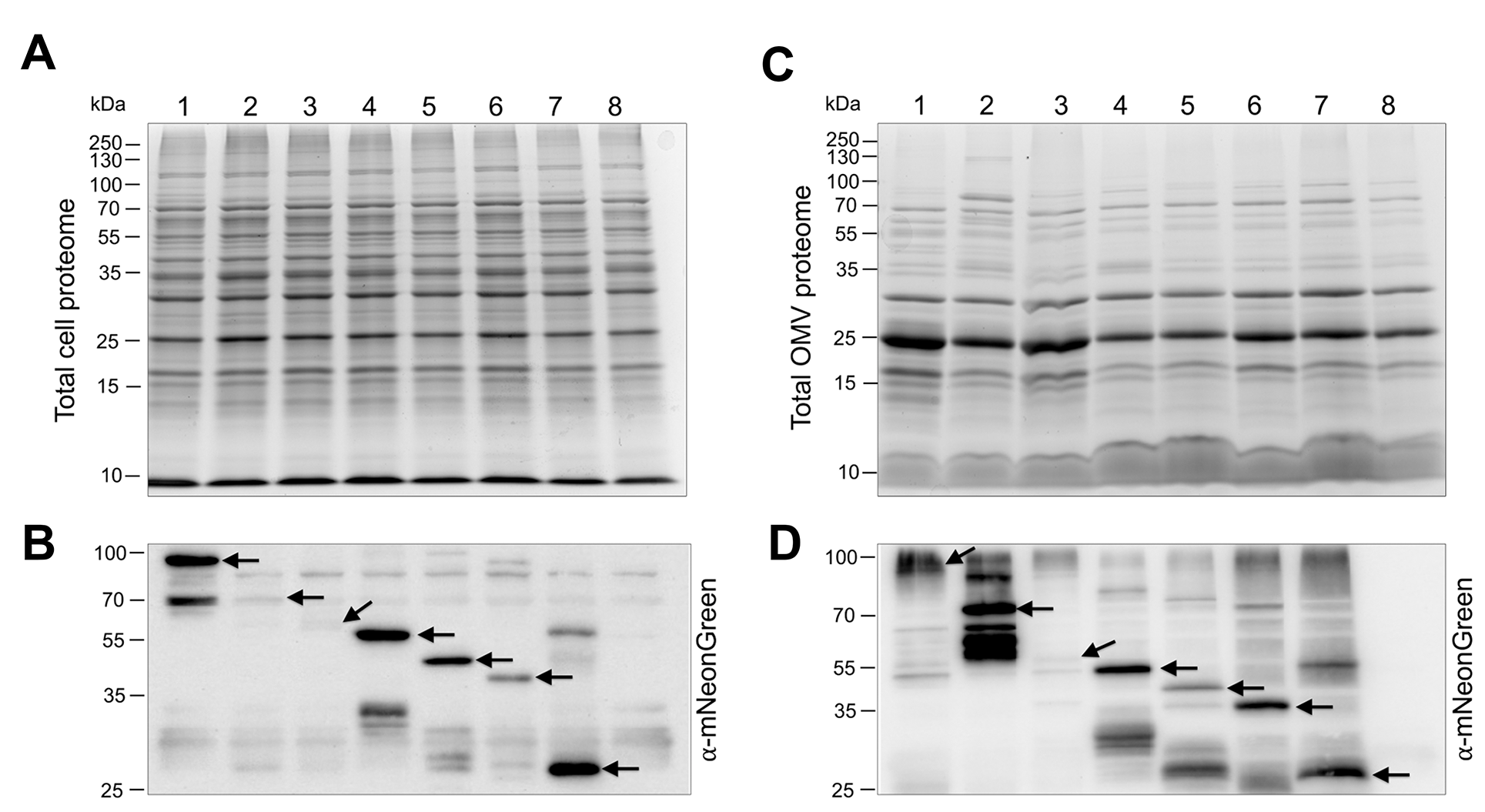


FIG S4 SDS-PAGE and western blot analysis of the total proteome of cells and OMVs from strains expressing various truncated *lpoA* variants fused to the reporter mNeonGreen*.* Total cell lysate (A and B) and OMV sample (C and D) obtained from *H. influenzae* RD carrying plasmids that encode for LpoA^1-576^-mNeonGreen (lane 1, predicted size 88 kDa), LpoA^1-471^-mNeonGreen (lane 2, predicted size 76 kDa), LpoA^1-361^-mNeonGreen (lane 3, predicted size 64 kDa), LpoA^1-256^-mNeonGreen (lane 4, predicted size 53 kDa), LpoA^1-193^-mNeonGreen (lane 5, predicted size 46 kDa), LpoA^1-124^-mNeonGreen (lane 6, predicted size 38 kDa), LpoA^1-26^-mNeonGreen (lane 7, predicted size 27 kDa) and the negative control P4-mCherry (lane 8). The samples were loaded in 12% SDS-PAGE gels in denaturing conditions (A and C) and transferred o/n to PVDF membranes on which western blots were performed (B and D). Rabbit polyclonal ⍺-mNeonGreen (1:10,000 dilution) was used to detect the constructs. Black arrows indicate the LpoA-mNeonGreen variants.
