# Supplementary material for: Protein domain-dependent vesiculation of Lipoprotein A, a protein that is important in cell wall synthesis and fitness of the human respiratory pathogen Haemophilus influenzae
Source: Front Cell Infect Microbiol. 2022 Oct 7;12:984955. doi: 10.3389/fcimb.2022.984955 (PMC9585305; doi:10.3389/fcimb.2022.984955)
Supplement: Supplementary file 5 [file DataSheet_5.docx]

**Figure S5, Jalalvand *et al.***

**A** **B**


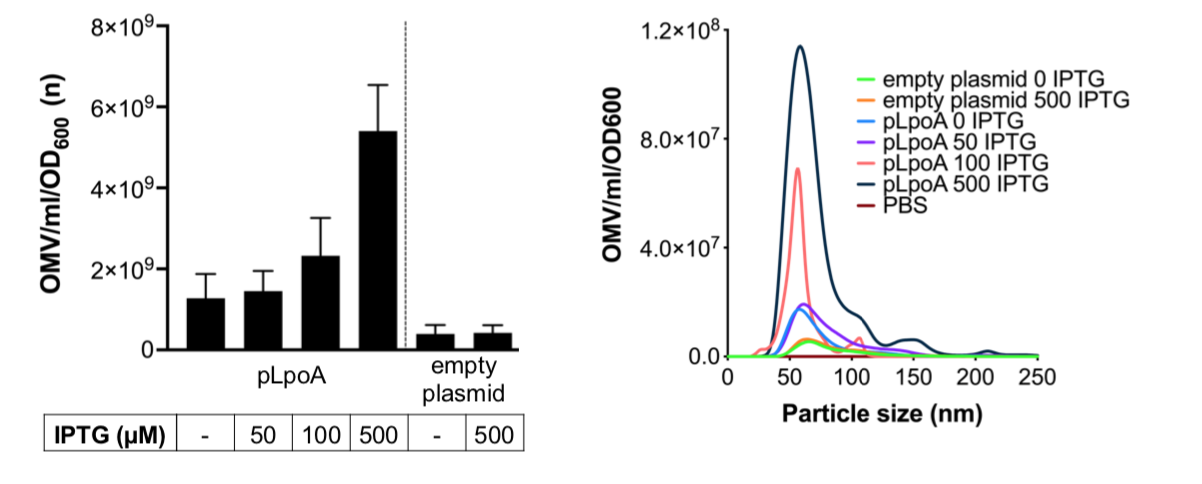


FIG S5 Overexpression of native LpoA causes hypervesiculation. NTA analysis shows quantification (A) and the size distribution (B) of OMVs purified from *H. influenzae* Rd carrying pLpoA or an empty plasmid control cultured in BHI with the indicated IPTG concentrations.

**Overexpression of LpoA causes hypervesiculation**

As LpoA is vital for bacterial fitness, we hypothesized that the removal of the lipoprotein via OMVs may be a strategy to regulate its cellular concentration. We therefore overexpressed full length non-tagged LpoA from a plasmid using the *lac*-promoter (pLpoA) in *H. influenzae* Rd to study the effect on cellular fitness and vesiculation. Empty plasmid was used as a control. OMVs were purified from overnight (o/n) stationary phase broth cultures supplemented with various concentrations of IPTG (0-500 µM), and NTA-quantification was performed (Fig SX). Bacterial cultures with the empty plasmid produced 3.94 x 10^8^ ± 2.21 x 10^8^ OMVs/ml/OD_600nm_ (without IPTG) and 4.22 x 10^8^ ± 1.88 x 10^8^ OMVs/ml/OD_600nm_ (with 500 µM IPTG), respectively. In contrast, the transformant carrying functional pLpoA produced 5.40 x 10^9^ ± 1.14 x 10^9^ OMVs/ml/OD_600nm_ when grown in the presence of 500 µM IPTG.
