# Supplementary material for: Protein domain-dependent vesiculation of Lipoprotein A, a protein that is important in cell wall synthesis and fitness of the human respiratory pathogen Haemophilus influenzae
Source: Front Cell Infect Microbiol. 2022 Oct 7;12:984955. doi: 10.3389/fcimb.2022.984955 (PMC9585305; doi:10.3389/fcimb.2022.984955)
Supplement: Supplementary file 6 [file DataSheet_6.docx]

**Supplementary Fig. S6, Jalalvand *et al.***


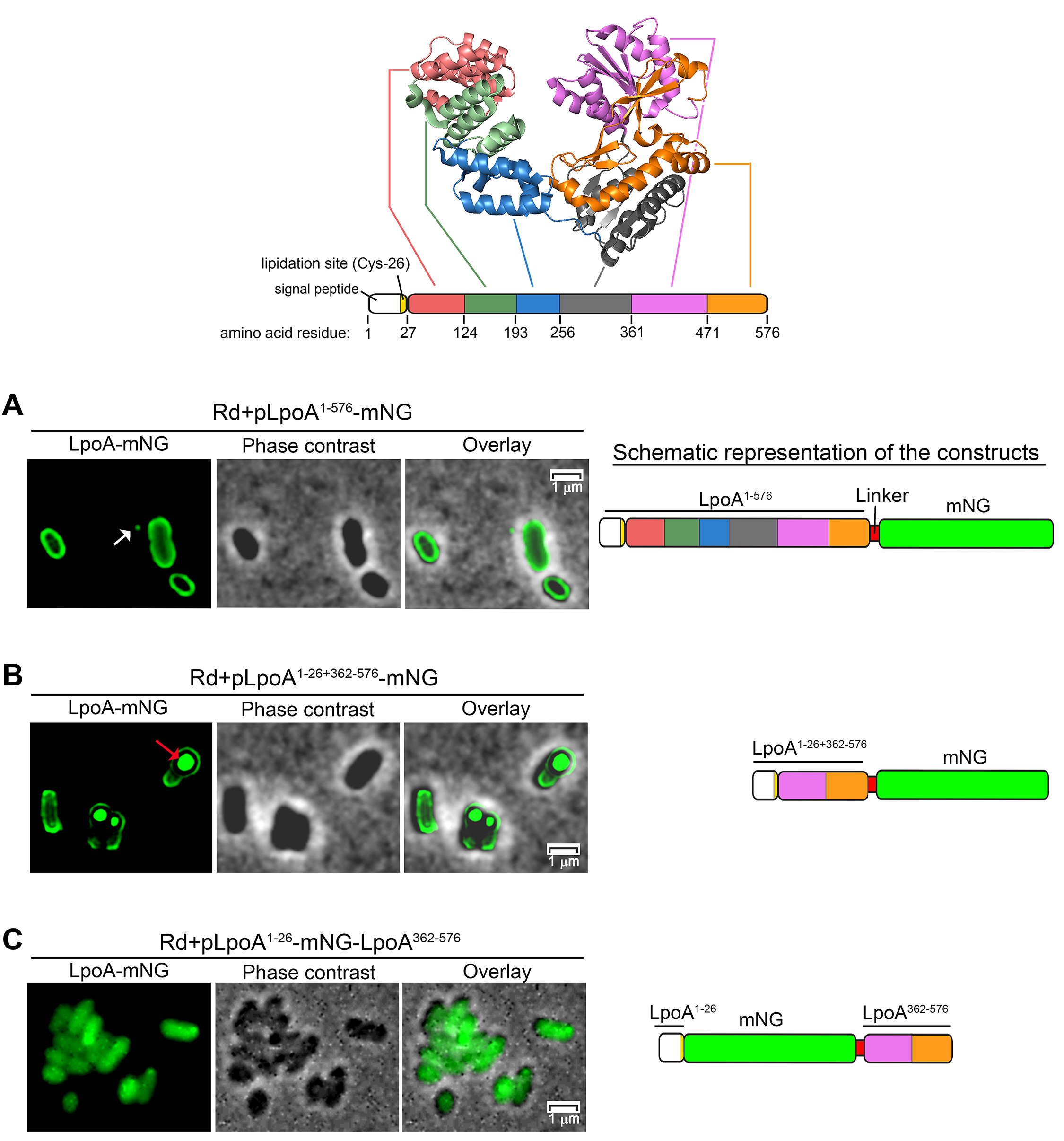


FIG S6 Phase contrast and fluorescence microscopy of *H. influenzae* Rd producing the LpoA C-terminus fused to the reporter mNeonGreen*.* Bacteria were grown in CDM supplemented with 100 µM IPTG. Microscopy was performed of bacteria in early stationary phase. While to the full length LpoA was succesfully produced and exported to the OM and OMVs (white arrow) **(**A), the C-terminal constructs would either make inclusion bodies (red arrow) (B) or be trapped in the cytoplasm (C). Schematic representations of the proteins are seen on the right.
