# Supplementary material for: Protein domain-dependent vesiculation of Lipoprotein A, a protein that is important in cell wall synthesis and fitness of the human respiratory pathogen Haemophilus influenzae
Source: Front Cell Infect Microbiol. 2022 Oct 7;12:984955. doi: 10.3389/fcimb.2022.984955 (PMC9585305; doi:10.3389/fcimb.2022.984955)
Supplement: Supplementary file 7 [file Table_1.docx]

**Supplementary Table S1, Jalalvand *et al.***

**TABLE S1** Strains and plasmids used in this study.

| **Strain** | **Genotype** | **Plasmid**  (induced by IPTG) | **Source** |
| --- | --- | --- | --- |
| *H. influenzae* 3656 | Wild-type |  | Laboratory collection |
| *H. influenzae* 3655 Δ*lpoA* | Δ*lpoA::cat* |  | This study |
| *H. influenzae* Rd | Wild-type |  | Laboratory collection |
| *H. influenzae* Rd P4^cherry^ | *omp_P4::omp_P4-mCherry-cat* |  | This study |
| *H. influenzae* Rd pLpoA^1-576^-mNG+ P4^mcherry^ | *lpoA::lpoA-mNeonGreen-cat* | pP4-mCherry | This study |
| *H. influenzae* Rd P4^cherry^+pLpoA^1-576^-mNG | *omp_P4::omp_P4-mCherry-cat* | pLpoA^1-576^-mNeonGreen | This study |
| *H. influenzae* Rd P4^mcherry^+pLpoA^1-471^-mNG | *omp_P4::omp_P4-mCherry-cat* | pLpoA^1-471^-mNeonGreen | This study |
| *H. influenzae* Rd P4^mcherry^+pLpoA^1-361^-mNG | *omp_P4::omp_P4-mCherry-cat* | pLpoA^1-361^-mNeonGreen | This study |
| *H. influenzae* Rd P4^mcherry^+pLpoA^1-256^-mNG | *omp_P4::omp_P4-mCherry-cat* | pLpoA^1-256^-mNeonGreen | This study |
| *H. influenzae* Rd P4^mcherry^+pLpoA^1-193^-mNG | *omp_P4::omp_P4-mCherry-cat* | pLpoA^1-193^-mNeonGreen | This study |
| *H. influenzae* Rd P4^mcherry^+pLpoA^1-124^-mNG | *omp_P4::omp_P4-mCherry-cat* | pLpoA^1-124^-mNeonGreen | This study |
| *H. influenzae* Rd P4^mcherry^+pLpoA^1-26^-mNG | *omp_P4::omp_P4-mCherry-cat* | pLpoA^1-26^-mNeonGreen | This study |
| *E. coli* DH5α | Wild-type |  | Laboratory collection |
| *E. coli* BL21(DE3) | Wild-type |  | Laboratory collection |
| *E. coli* JKE201 | Wild-type |  | ^1^ |
|  |  |  |  |
|  |  |  |  |
| **Plasmids (laboratory collection designation)** | **Construct** |  | **Source** |
| pLpoA (pFJLU29) | P_lac_::*lpoA* (full length), the plasmid backbone for all plasmids unless stated otherwise is pBZ485^1^ |  | This study |
| pLpo^1-576^-mNeonGreen (pFJLU25) | P_lac_::*lpoA-mNeonGreen* (full length) |  | This study |
| pLpoA^1-471^-mNeonGreen (pFJLU39) | P_lac_::*lpoA*^(aa 1-471)^*-mNeonGreen* |  | This study |
| pLpoA^1-361^-mNeonGreen (pFJLU38) | P_lac_::*lpoA*^(aa 1-361)^*-mNeonGreen* |  | This study |
| pLpoA^1-256^-mNeonGreen (pFJLU37) | P_lac_::*lpoA*^(aa 1-256)^*-mNeonGreen* |  | This study |
| pLpoA^1-193^-mNeonGreen (pFJLU36) | P_lac_::*lpoA*^(aa 1-193)^*-mNeonGreen* |  | This study |
| pLpoA^1-124^-mNeonGreen (pFJLU35) | P_lac_::*lpoA*^(aa 1-124)^*-mNeonGreen* |  | This study |
| pLpoA^1-26^-mNeonGreen (pFJLU34) | P_lac_::*lpoA*^(aa 1-26)^*-mNeonGreen* |  | This study |
| pP4-mCherry (pFJLU32) | P_lac_::*omp_P4-mCherry* (full length) |  | This study |
| pLpoA-periplasmic (pFJLU26) | P_lac_::*fbpA*^(aa 1-29)^*-* *lpoA*^(aa 27-576)^ |  | This study |
| pLpoA-C-term1 (pFJLU42) | P_lac_::*lpoA*^(aa 1-26 + 362-576)^*-mNeonGreen* |  | This study |
| pLpoA-C-term2 (pFJLU43) | P_lac_::*lpoA*^(aa 1-26)^*-mNeonGreen- lpoA*^(aa 362-576)^ |  | This study |
| pET26-mNeonGreen | P_T7_::*mNeonGreen* (full length, aa 1-237) |  | This study |

**Reference**

1 Harms, A. *et al.* A bacterial toxin-antitoxin module is the origin of inter-bacterial and inter-kingdom effectors of *Bartonella*. *PLoS Genet* **13**, e1007077, doi:10.1371/journal.pgen.1007077 (2017).
