# Supplementary material for: Protein domain-dependent vesiculation of Lipoprotein A, a protein that is important in cell wall synthesis and fitness of the human respiratory pathogen Haemophilus influenzae
Source: Front Cell Infect Microbiol. 2022 Oct 7;12:984955. doi: 10.3389/fcimb.2022.984955 (PMC9585305; doi:10.3389/fcimb.2022.984955)
Supplement: Supplementary file 8 [file Table_2.docx]

**Supplementary Table S2, Jalalvand *et al.***

**TABLE S2** Nanotrack analysis of the size distribution of OMVs obtained at various time points during the bacterial growth curve. The mean, mode (the peak of the highest curve), standard deviation (SD), 10^th^ (D10), 50^th^ (D50) and 90^th^ (D90) percentile are stated.

| **Sample** | **Mean (nm)** | **Mode (nm)** | **SD (nm)** | **D10 (nm)** | **D50 (nm)** | **D90 (nm)** |
| --- | --- | --- | --- | --- | --- | --- |
| 5 h | 93.0 | 66.1 | ±36.8 | 58.8 | 82.1 | 142.0 |
| 8 h | 75.2 | 57.8 | ±30.3 | 49.3 | 64.6 | 118.4 |
| 10 h | 76.0 | 55.2 | ±32.2 | 46.7 | 65.9 | 122.7 |
| 12 h | 75.4 | 54.3 | ±30.6 | 48.7 | 67.1 | 115.4 |
| 14 h | 80.5 | 60.0 | ±35.3 | 51.8 | 68.5 | 126.3 |
| 16 h | 75.7 | 56.1 | ±30.0 | 51.0 | 66.1 | 114.5 |
| 18 h | 71.6 | 55.9 | ±24.9 | 50.5 | 63.7 | 102.3 |
| 20 h | 78.1 | 60.3 | ±31.9 | 51.4 | 68.3 | 117.1 |
